# Structural control of self-assembled peptide nanostructures to develop peptide vesicles for photodynamic therapy of cancer

**DOI:** 10.1016/j.mtbio.2022.100337

**Published:** 2022-06-22

**Authors:** Soo hyun Kwon, Donghyun Lee, Hyoseok Kim, You-jin Jung, Heebeom Koo, Yong-beom Lim

**Affiliations:** a Department of Materials Science and Engineering, Yonsei University, Seoul, 03722, Republic of Korea; b Department of Medical Life Sciences and Department of Biomedicine & Health Sciences, College of Medicine, The Catholic University of Korea, Seoul, 06591, Republic of Korea

**Keywords:** Convergent correlation, Self-assembly, Peptides, Morphology, Photodynamic therapy, Peptidesome

## Abstract

Vesicles such as liposomes, polymersomes, and exosomes have been widely used as drug delivery carriers; however, peptide vesicles (peptidesomes) despite their potential utility are far less well developed. Peptidesomes are distinctive because peptides play dual roles as a self-assembly building block and a bioactive functional unit. In order for peptidesomes to become successful nanodrugs, the issues related to differences in nanostructural properties between *in vitro* and *in vivo* conditions should be addressed. Here, we delineate a multivariate approach to feedback control the structures of peptide building blocks, nanoparticle size, drug loading process, nanoparticle aggregation, cytotoxicity, cell targeting capability, endosome disruption function, protease resistance, and *in vivo* performance, which eventually enabled the successful development of a highly efficacious peptidesome for *in vivo* cancer therapy. This study lays the groundwork for the successful *in vivo* translation of peptide nanodrugs.

## Graphical abstract

**Figure:**
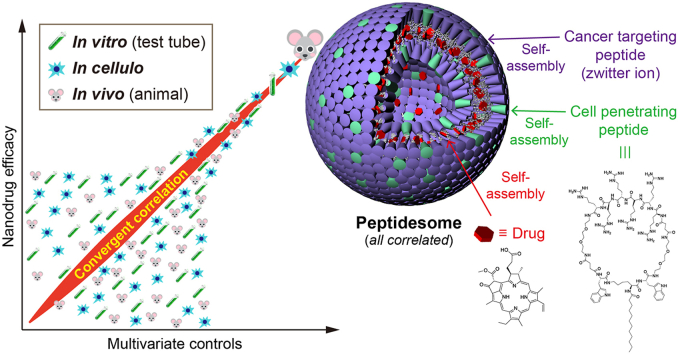

## Credit author statement

Yong-beom Lim: Conceptualization, Writing – original draft, Writing – review & editing, Supervision, Project administration, Funding acquisition, Heebeom Koo: Conceptualization, Writing – original draft, Writing – review & editing, Supervision, Project administration, Funding acquisition, Soo hyun Kwon: Methodology, Software, Validation, Formal analysis, Investigation, Resources, Data curation, Writing – original draft, Writing – review & editing, Visualization, Donghyun Lee: Methodology, Software, Validation, Formal analysis, Investigation, Resources, Data curation, Writing – original draft, Writing – review & editing, Visualization, Hyoseok Kim: Methodology, Software, Validation, Formal analysis, Investigation, Resources, Data curation, Writing – original draft, Writing – review & editing, Visualization, You-jin Jung: Investigation, Writing – original draft, Writing – review & editing, Visualization.

## 1 Introduction

Interests in self-assembled peptide nanostructures (SPNs) has been escalated in recent years. SPNs have been utilized in applications ranging from sensing and catalysts to therapeutics [[1], [2], [3]]. In particular, SPNs are well-suited for biorelated applications considering that their constituent amino acids are bioderived and biocompatible molecules. Similar to proteins, a wide variety of 2D and 3D structures can be fabricated by simply changing the amino acid sequences. Even further, chemical modifications and the adoption of unique molecular topologies such as cyclic and dendritic structures in peptide supramolecular building blocks can increase the nanostructural and functional diversity of SPNs [4,5].

Vesicles are among the most widespread drug carrier applications of self-assembled nanostructures [6]. As building blocks for self-assembly, molecules based on lipids and synthetic polymers [7] that respectively assemble into liposomes and polymersomes have been the most widely used in drug delivery. In comparison, peptide building blocks are far less developed, and examples of medical translation are scarce, in part due to their relatively short history. In a practical sense, most of the medical advancements in nanodrugs have been made with lipid building blocks rather than with synthetic polymers and peptide building blocks. For example, one of the most famous vesicular drug delivery carriers such as Doxil [6] is based on lipids. Lipids are also major components of exosomes or other extracellular vesicles and have recently drawn significant attention as potential drug carriers [8].

Because nanomaterials made from synthetic polymers and peptides have their own advantageous properties and unique functions, they might be able to become highly sophisticated and successful drug delivery system (DDS) once the potential hurdles encountered during their development can be overcome. In the case of SPNs, particularly less attention has been given to correlating *in vitro* test tube results with *in cellulo* and *in vivo* studies. Because peptide self-assembly behaviors and bioactivity can differ considerably between those conditions, this issue must be resolved for the successful translation of basic research into clinical practice.

Here, we report our systematic approaches aimed at convergently correlating *in vitro*, *in cellulo*, and *in vivo* properties of nanoassemblies during the development of DDS based on peptidesomes and describe our heuristic solutions to the number of potential hurdles and pitfalls. Notably, we found that special care should be taken when correlating the morphology and size of SPNs, two of the most important structural characteristics for nanomaterials, among *in vitro*, *in cellulo*, and *in vivo* studies. We subdivided several critical considerations for SPN DDS development as follows (i) the selection of peptide building blocks and nanoscale size issues, (ii) drug loading can initiate morphological transformation and superstructure formation, (iii) an inversely proportional relationship between intracellular delivery efficiency and cytotoxicity, (iv) the prevention of large aggregate formation under *in vivo* conditions, and (v) the necessary conditions for successful *in vivo* therapy with SPN nanodrugs. We hope that this attempt will provide useful guidelines for the development of SPN therapeutics and DDS in general. In addition, this study demonstrates that peptidesomes assembled from cyclic peptide building blocks can be developed as promising nanodrugs for solid cancer.

## 2 Experimental section

### 2.1 General

General chemicals were obtained from Sigma-Aldrich (USA) and Merck (Germany). Fmoc-amino acids and coupling reagents were purchased from Novabiochem (Germany) and Anaspec (USA). The oligoethylene glycol-based linker N-(Fmoc-8-amino-3,6-dioxaoctyl)succinamic acid (Fmoc-PEG2-Suc-OH or Fmoc-Ebes-OH) was purchased from Anaspec. HPLC solvents and media were purchased from Fisher Scientific (USA). Pheophorbide a was purchased from Cayman (USA). Thiazoyl blue tetrazolium bromide (MTT) was purchased from Biosesang (Seongnam, Gyeonggi-do, Korea). Hoechst 33,342 was obtained from Thermo Fisher Scientific (Waltham, MA, USA). The sizes of self-assembled nanoparticles were analyzed by a dynamic light scattering size distributor (Particle Size & Zeta Potential Analyzer, ELS-1000ZS, Otsuka Electronics, Japan) using a 1 ​cm path length UV-transparent cuvette. The secondary structures of the cyclic peptide building blocks in SPN were measured using a Chirascan circular dichroism spectrometer equipped with a Peltier temperature controller (Applied Photophysics, UK). The circular dichroism (CD) spectra of the samples were analyzed from 260 to 190 ​nm using a 2 ​mm path length cuvette. The molar residue ellipticity of the sample was calculated per amino acid residue. All mouse experiments were conducted under an animal protocol approved by The Catholic University of Korea on Laboratory Animal Care (2020-0359-05).

### 2.2 Peptide syntheses, head-to-tail cyclization, and conjugation

The first residue (Fmoc-Ebes-OH) was loaded on 2-chlorotrityl resin (Novabiochem, Germany) in 1 ​M diisopropylethylamide (DIPEA)/methylene chloride (MC). Coupling of the following amino acids was performed using standard Fmoc protocols in a Tribute peptide synthesizer (Protein Technologies, USA). Standard amino acid protecting groups were used for the synthesis except Dde-Lys (Fmoc)-OH. To prepare the protected peptide fragment, the N-terminal Fmoc group was removed, followed by treatment of the peptide-loaded resin with a cleavage cocktail of acetic acid/2,2,2-trifluoroethanol (TFE)/MC (2:2:6, v/v/v) for 1–2 ​h, and the filtrate was collected (4 ​mL ​× ​2 cycles). Acetic acid was removed as an azeotrope with hexane to obtain a white powder. Pseudo-high-dilution conditions for head-to-tail cyclization were achieved using a dual syringe method. One syringe was filled with the protected peptide fragment (20 ​μmol, 1 equiv.) and DIPEA (4 equiv.) in DMF (20 ​mL), while the other syringe was filled with 2-(6-chloro-1*H*-benzotriazole-1-yl)-1,1,3,3-tetramethylaminium hexafluorophosphate (HCTU, 1 equiv.) in DMF (20 ​mL). The solutions in both syringes were added to a round bottomed flask containing HCTU (0.1 equiv.) and hydroxybenzotriazole (HOBt, 1 equiv.) in DMF (20 ​mL) at a rate of 0.06 ​mL/min using a syringe pump while stirring. The reaction mixture was further stirred overnight at 55 ​°C after the completion of the syringe injection. Then, DMF was removed by rotary evaporation, and the peptide was precipitated using a mixture of MC, *tert*-butyl methyl ether (TBME), and hexane. The Dde group in lysine was orthogonally deprotected using 2% (v/v) hydrazine/DMF (2 ​min ​× ​4 cycles).

To conjugate a hydrocarbon tail, the cyclized peptide fragment (20 ​μmol, 1 equiv.), lauric acid (5 equiv.), and DIPEA (10 equiv.) were dissolved in DMF (2 ​mL) and shaken overnight. For the conjugation of pheophorbide a (**Pa**), a succinimidyl ester (NHS ester) of **Pa** was first prepared by the reaction of **Pa** (20 ​mg, 1 equiv), *N*-hydroxysuccinimide (NHS, 1.7 equiv.), 1-ethyl-3-(3-dimethylaminopropyl)carbodiimide hydrochloride (EDC, 1.7 equiv.), and 4-dimethylaminopyridine (DMAP, 0.4 equiv.) in MC (6 ​mL) in the dark overnight. The product was precipitated with distilled water (DW) and recovered by centrifugation. The cyclized peptide fragment (25 ​μmol, 1 equiv.), NHS ester of **Pa** (7 equiv.), triethylamine (14 equiv.), EDC (14 equiv.), and DMAP (14 equiv.) were dissolved in MC (5 ​mL) and shaken for 2 days. Following the evaporation of MC, the product was redissolved in a small volume of MC and precipitated using a mixture of TBME and hexane. The final deprotection was performed in a cleavage cocktail (TFA/TIS/water; 95:2.5:2.5, v/v/v) for 3 ​h, followed by trituration with TBME. The cyclic peptide building blocks were purified by reversed-phase high-performance liquid chromatography (HPLC) using water (0.1% TFA) and acetonitrile (0.1% TFA) as eluents. The molecular weight of the peptide was confirmed by matrix-assisted laser desorption/ionization time-of-flight (MALDI-TOF) mass spectrometry. The purity of the peptide was >95%, as judged by analytical HPLC. The product concentration was determined spectrophotometrically in water/acetonitrile (1:1) using the molar extinction coefficient of tryptophan (5502 ​M^−1^ ​cm^−1^) at 280 ​nm and **Pa** (44,500 ​M^−1^ ​cm^−1^) at 667 ​nm for **R**_**n**_ and **R**_**6**_**-Pa**, respectively.

### 2.3 Self-assembly and drug loading

Typically, cyclic peptide building blocks were initially dissolved in 30% hexafluoroisopropanol (HFIP) in water (v/v) to promote disassembly and molecular mixing. Then, the solvents were evaporated, and the peptide was rehydrated with an appropriate solvent or buffer. A similar procedure was conducted for drug loading. Briefly, both cyclic peptide building blocks and **Pa** were dissolved in 30% HFIP in water (v/v), followed by the solvent evaporation and rehydration.

### 2.4 Atomic force microscopy (AFM)

Five microliters samples were placed onto a freshly cleaved mica surface and dried. When the salt was present in the specimen, the excess salt was removed by washing the mica with 3 ​μL of DW. Then, the excess water was wicked off, and the mica was quickly dried under a stream of argon. The specimen was analyzed using an NX10 AFM instrument (Park Systems, Korea) in noncontact mode. Scan rate was 1.0 ​Hz. The data were analyzed using XEN software.

### 2.5 Transmission electron microscopy (TEM)

Three microliters samples were placed onto a carbon-coated copper grid. After 1 ​min, the excess sample was wicked off by a filter paper. For negative staining, a 1–2 ​μL drop of 0.1% (w/v) uranyl acetate/distilled water was added to the grid. After 1 ​min, the excess staining solution was wick off by a filter paper. The specimen was analyzed using a JEM-F200 field emission transmission electron microscope (JEOL, Japan) at an accelerating voltage of 200 ​kV. The data were analyzed using GATAN software.

### 2.6 *In vitro* cellular uptake and FACS analysis

Cellular uptake of materials in SCC7 cells was analyzed based on the intrinsic fluorescence of **Pa** via CLSM (LSM700, Carl Zeiss, Germany) and flow cytometry (FACS Canto II, BD Biosciences, Bedford, MA, USA). SCC7 cells were seeded in 24-well plates at a density of 5 ​× ​10^4^ and incubated overnight. Then, 200 ​μL of the sample in an appropriate solution was added to 800 ​μL of complete medium for each well. The cells were treated with the samples for 4 ​h followed by the removal of the sample solution and the washing step. For cell imaging, nuclei were also stained with Hoechst 33,342.

### 2.7 Detection of ROS generation

In vitro and *in vivo* ROS production was measured using 2′,7′-dichlorofluorescin diacetate (DCFDA). SCC7 cells were treated with 2 μg/ml of peptidesome←**Pa** (G&G) for 1 ​h and washed out. After that, the cells were treated with 20 ​μm of DCFDA in PBS for 30 ​min and then laser irradiated to the cells. DCFDA fluorescence of the cells was measured in the FITC wavelength by flow cytometry. For *in vivo* ROS measurement, 50 ​mg/kg of DCFDA was intratumorally injected into SCC7 tumor-bearing mice and then PDT was performed as previously reported [9]. Tumor tissue was excised from the mice and cryosections were prepared with a thickness of 10 ​μm, followed by detecting fluorescence of DCFDA in inverted fluorescence microscopy.

### 2.8 *In vivo* biodistribution analysis

SCC7 tumor-bearing mice were developed by injecting 2 ​× ​10^6^ ​cells in 30 ​μL of saline subcutaneously into the left thigh of C3H/HeN mice. When the tumor size reached 200–250 ​mm^3^, 100 ​μL of the sample solution was administered via the tail vein. Whole-body biodistribution was observed at 3, 6, 12, and 24 ​h after the injection of the samples using IVIS Lumina XRMS (PerkinElmer, Inc., Waltham, MA, USA). At each time point, 30 ​μL of blood was collected from the tail vein, and the fluorescence imaging was performed by an IVIS system. At the last time point, the tumor and major organs (heart, lung, liver, spleen, and kidney) were dissected, and *ex vivo* fluorescence images were obtained by IVIS. IVIS imaging was performed at a wavelength of Cy5.5. The dissected tumors were fixed in 4% paraformaldehyde for 24 ​h and treated with increasing concentrations from 10% sucrose to 20% sucrose. Then, tumor tissues were frozen in optimal cutting temperature (OCT) compound and sectioned at 10 ​μm thickness. Sectioned tissues were attached to glass slides and dried. The tissues were washed several times in PBS and counterstained with 2 ​μg/mL of Hoechst 33,342 for 20 ​min at room temperature. The fluorescence from the tissue was observed in an Observer. Z1 inverted fluorescence microscope (Carl Zeiss, Jena, Germany).

### 2.9 *In vivo* antitumor efficacy measurement

The SCC7 tumor-bearing mouse models were prepared similarly to the *in vivo* imaging experiment, and each sample was prepared in the same way as in the *in vivo* IVIS imaging experiments. The samples were intravenously injected into the mice when the tumor volume reached approximately 50 ​mm^3^. Three hours postinjection of the samples, NIR laser (671 ​nm) irradiation was administered to the tumor site with a power of 0.53 ​W/cm^2^ for 15 ​min, and the same sample injection and NIR irradiation procedures were repeated at the next day. The tumor volume and body weight were recorded every two days. At the end of the therapy, major organs and tumors were dissected for histological analysis. They were fixed, sliced, stained with haematoxylin and eosin (H&E), and observed with a microscope (AxioImager A1, Zeiss, Germany).

### 2.10 Statistical analysis

Student's t-test was used to compare the differences between the two groups. One-way analysis of variance (ANOVA) and Tukey's post hoc analysis were used to compare differences between multiple groups. A value of P ​< ​0.05 was considered statistically significant.

## 3 Results

### 3.1 Selection of peptide building blocks and nanoscale size issues

In most cases, supramolecular building blocks, including lipids, polymers, and peptides, assemble into common morphologies, i.e., spherical micelles, cylindrical micelles, and vesicles. Among them, vesicles have been used most extensively as drug delivery carriers. Intracellular vesicular transport systems, exosomes, and even enveloped viruses are also vesicles in terms of self-assembled morphology. One of the advantageous features of vesicles is that drugs can be loaded using two different mechanisms. Nonpolar drugs are more likely to be incorporated in the hydrophobic space of the vesicular bilayer via an encapsulation mechanism, whereas relatively polar drugs are more likely to be incorporated in the water-filled interior space of the vesicle via an entrapment mechanism. In contrast, spherical and cylindrical micelles can employ only encapsulation mechanisms for drug loading. In particular, the loading of drugs by the encapsulation mechanism is especially suitable for polymers and peptides because they can form more robust nanoparticles than lipids [10,11]. Given the usefulness of vesicles in drug delivery and the multifunctionality of peptides as supramolecular building blocks, we intend to design and fabricate peptidesomes that can become highly functional nanodrugs *in vivo*.

In designing peptide building blocks, we placed particular emphasis on the following criteria. First, peptide building blocks should have a strong propensity to form vesicles rather than other morphologies, which would facilitate performing feedback control of the SPN nanostructural properties by modifying the chemical structures of the peptides. We elected to use cyclic peptides as building blocks because peptides that are both cyclic and amphiphilic have shown a strong tendency to self-assemble into vesicles (peptidesomes) [11]. Second, the hydrophilic segment of the building blocks would contain a large proportion of arginine residues for the efficient intracellular translocation of SPNs. In many cell penetrating peptides, arginine plays a key role in cell surface attachment and membrane translocation processes [12,13]. Third, to achieve high efficacy *in vivo*, peptidesomes should be sufficiently small (preferably, ≤ 100 ​nm) [14,15]. Fourth, the building blocks should be as simple in structure and as low in molecular weight as possible because large-scale production with high purity is a very important issue in drug development. In the case of cyclic peptides, the synthetic yield drops rapidly as the ring sizes increase.

**Fig. 1:**
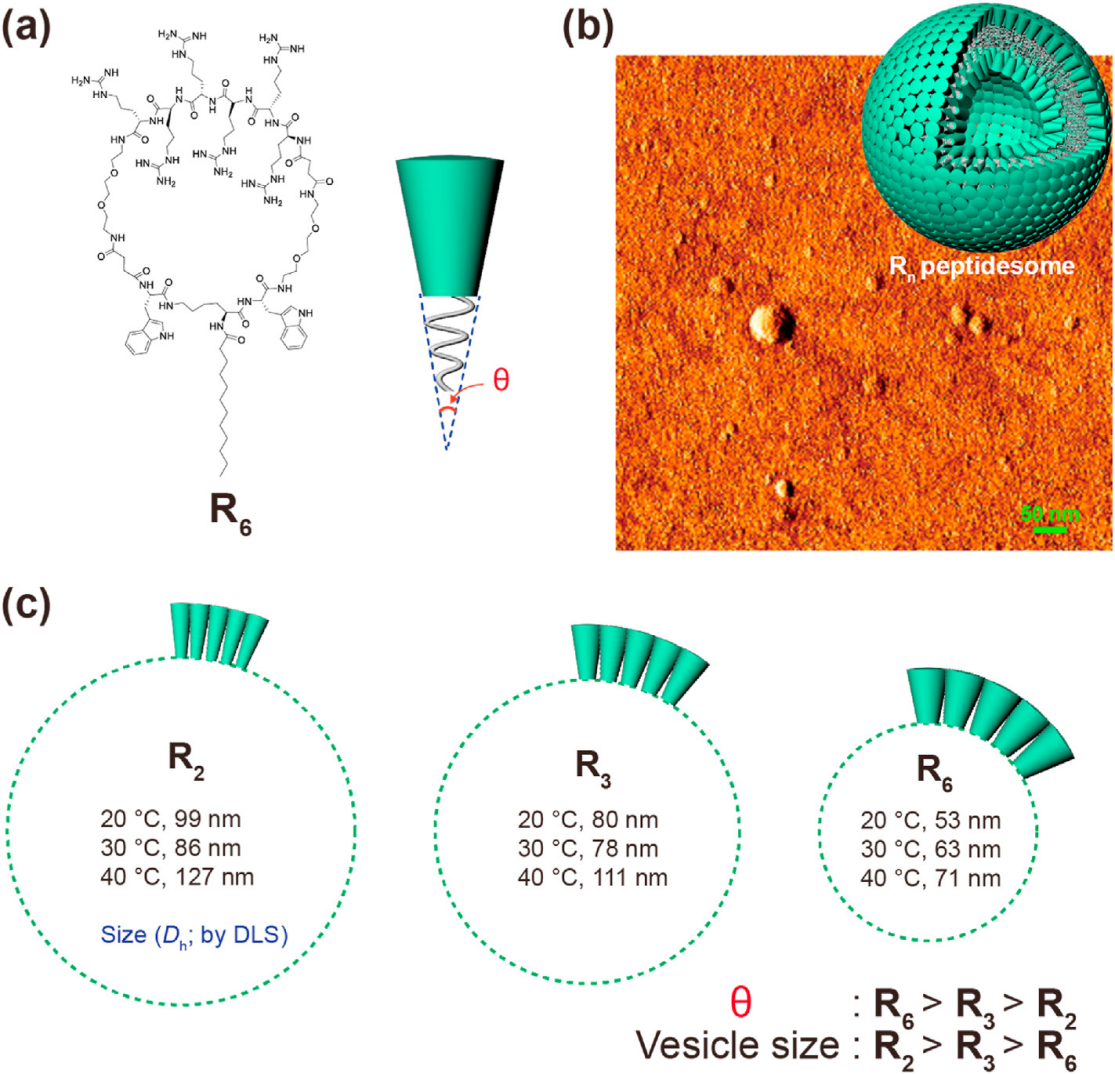
Peptidesome assemblies. (a) Cyclic peptide building blocks. (b) AFM image of **R**_**2**_ and Model of **R**_**n**_ peptidesome. (c) Dependence of vesicle size on the cone angle of cyclic peptide building blocks. DLS experiments were performed in distilled water (DW).

We designed a cyclic peptide amphiphile **R**_**n**_ that has a hydrophilic segment with varying numbers of arginines and a hydrophobic segment consisting of two tryptophans and a C12 hydrocarbon (Fig. 1a and S1). Initially, the self-assembly behavior of the simplest one, **R**_**2**_ (two arginines), was investigated by dissolving it in DW, followed by probe sonication. Investigation of self-assembled morphology with atomic force microscopy (AFM) showed that **R**_**2**_ assembled into discrete spherical nanostructures (Fig. 1b and S4). The average hydrodynamic diameter (*D*_h_) measured with dynamic light scattering (DLS) ranged from 99 ​nm to 127 ​nm depending on the temperature (Fig. 1c and S5). Theoretically, spherical micelles are homogeneous in size, and their diameters are twice as large as the molecular length, whereas vesicles can be any size. Considering that the molecular length of a fully extended **R**_**2**_ is approximately 4.5 ​nm, the measured size of the spherical SPNs indicates that **R**_**2**_ assemblies are vesicles (peptidesome; Fig. 1b). Because the *D*_h_ (40 ​°C) near the body temperature (37 ​°C) was larger than 100 ​nm, we decided to modify the building block design. The self-assembled morphology of amphiphiles is influenced by the packing parameter (*P*) and molecular shape [16]. Simplifying the shape of the cyclic peptide amphiphile to a cone and anticipating that the cyclic building blocks would assemble into vesicles at a wide range of *P*, we expected that changes in the cone angle would have an influence on the final size of peptidesomes (Fig. 1c). We added more arginine residues to increase the volume fraction of the hydrophilic segments while maintaining the structure in the other part of the molecule (**R**_**3**_ and **R**_**6**_). Morphology of **R**_**3**_ and **R**_**6**_ were also confirmed to be discrete spherical nanostructures using AFM (Fig. S4). The TEM data of **R**_**6**_ further showed layer structure of spherical particles, and the thickness of layer was 10.26 ​± ​0.68 ​nm which well matched with the schematical structure of non-interdigitated bilayer model (∼9.92 ​nm) (Fig. S6). Therefore, we concluded self-assembled nanostructures of **R**_**n**_ were peptidesomes (vesicles). The general trend was that the size of peptidesomes decreased as more arginine residues were added. Because the **R**_**6**_ peptidesome was sufficiently smaller than 100 ​nm at all temperatures according to DLS data (Fig. 1c and S5), further development was performed with this building block.

### 3.2 Drug loading can initiate morphological transformation and superstructure formation

**Fig. 2:**
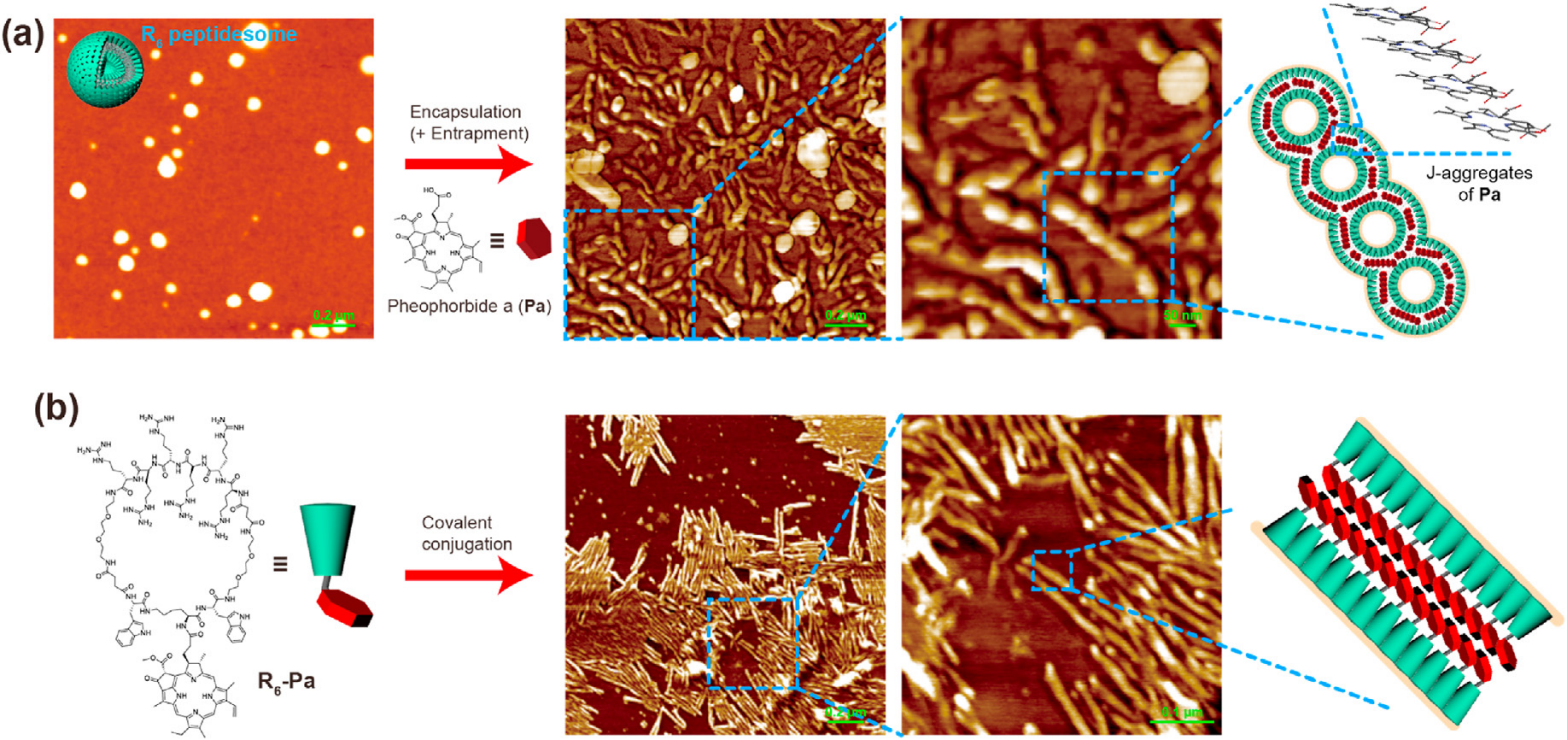
Drug loading and morphological transformation. (a) Morphological transformation into peapod-like elongated nanostructures after the loading of **Pa** in the **R**_**6**_ peptidesome. A large excess of **Pa** was used during the drug loading. (b) Self-assembly of a covalent conjugate of **R**_**6**_ and **Pa** (**R**_**6**_**-Pa**). All the experiments were performed in DW.

As stated in Lipinski's rule of five, most small molecule drugs are lipophilic overall [17]. Pheophorbide a (**Pa**), a small molecule drug used in this study, is also hydrophobic with a low water solubility of approximately 0.014 ​g/L. **Pa** is a porphyrin derivative of plant chlorophyll and has been used as a photosensitizer for photodynamic therapy (PDT) [18,19]. For noncovalent drug loading in **R**_**6**_ peptidesome, **R**_**6**_ and **Pa** were mixed in DW, and the solution was sonicated vigorously. After this drug loading process, the **R**_**6**_ peptidesomes underwent a morphological transformation into elongated superstructures, although some of the peptidesomes retained their original spherical morphologies (Fig. 2a). The thickness of the elongated superstructures coincided with those of adjacent vesicles, and many of them had the shape of peapods with an uneven surface, suggesting that the peapod-like superstructures were formed by the fusion of vesicles. Considering the hydrophobicity of **Pa** and the elongation behavior after drug loading, a major proportion of **Pa** molecules are likely to be encapsulated in the internal hydrophobic region of the bilayer, while a minor proportion of solvated **Pa** molecules are entrapped in the water-filled interior. Because of the strong hydrophobic properties of **Pa**, it was stably loaded into the peptidesome, and free **Pa** was not released from the peptidesome in PBS containing 2% (w/v) tween 80 ​at 37 ​°C for 24 ​h (Fig. S7).

A red-shift and an increased absorption intensity in the Q-band of **Pa** after the drug loading indicate the head-to-tail J-aggregation of the porphyrins within the peptidesome bilayer (Fig. S8) [20,21]. J-aggregation is generally described as staircase packing mode, while H-aggregation forms vertical stacks. Thus, the peptidesomes are likely connected and eventually fused by the head-to-tail stacking of **Pa** molecules (Fig. 2a). To further corroborate the elongation mechanism of the peptidesomes, we synthesized a peptide building block in which **Pa** is chemically conjugated to the **R**_**6**_ backbone (**R**_**6**_**-Pa**). **R**_**6**_**-Pa** assembled into elongated nanostructures whose shape was similar to that of the peapod-like nanostructure without an uneven surface, supporting the **Pa**-mediated fusion of **R**_**6**_ peptidesomes (Fig. 2b). The elongation behavior was observed only when the amount of **Pa** was in excess over that of **R**_**6**_. Taken together, the results demonstrate that we should be aware of the potential changes in nanostructural properties, such as morphological transformation and size increase, via the superstructure formation after the drug loading.

### 3.3 Inversely proportional relationship between intracellular delivery efficiency and cytotoxicity

**Fig. 3:**
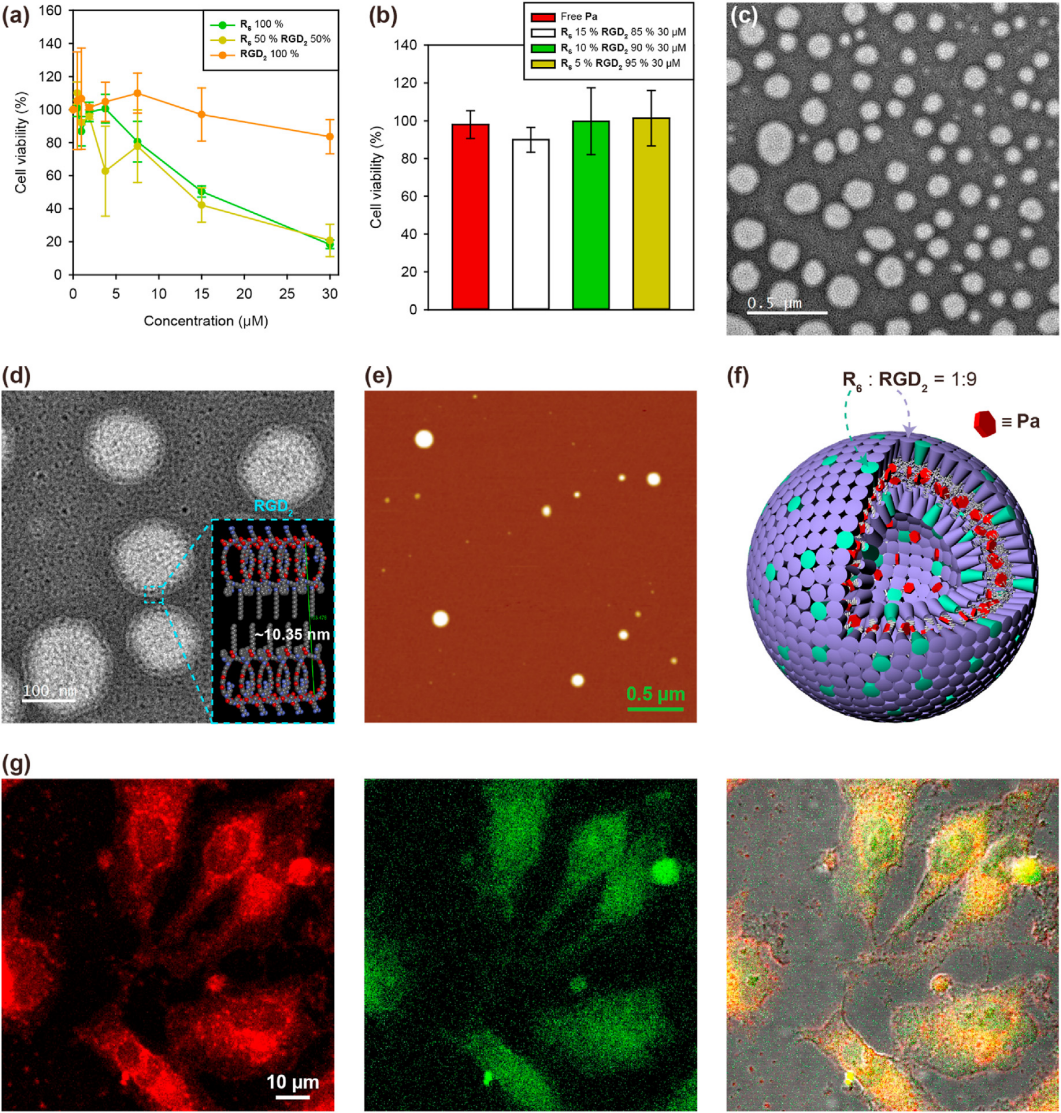
Balancing cell penetration and cytotoxicity. (a) Cytotoxicity of peptidesomes. (b) Fine-tuning of the **R**_**6**_ and **RGD**_**2**_ ratio to balance cell penetration efficiency and cytotoxicity. **Pa** loading ​= ​12 ​mol%. (c) TEM image of coassembled peptidesome (**R**_**6**_:**RGD**_**2**_ ​= ​1:9) (d) TEM image of coassembled peptidesome with schematical structure of bilayer. (e) AFM image of **Pa**-loaded peptidesome (i.e., peptidesome←**Pa**). (f) Schematic model of a peptidesome←**Pa**. (g) Confocal laser scanning microscopy (CLSM) image after the treatment of HeLa cells with the peptidesome←**Pa**. Right: **Pa** fluorescence (red). Middle: LysoTracker (green). Left: Overlay of fluorescence from **Pa** (red) and LysoTracker (green) merged with bright field image.

Every successful nanocarrier should have both high intracellular delivery efficiency and low toxicity; however, these two bioactivities can be mutually exclusive. During the cell entry processes, certain parts of the cell need to be abnormally disrupted to achieve a high intracellular delivery efficiency, which could result in the generation of toxic side effects. Because toxicity (safety) is the primary evaluation criterion in a phase I clinical trial, toxic nanocarriers should not be able to pass through this phase. We aimed to achieve a high intracellular delivery efficiency by making use of the arginine-rich surface of SPNs based on the fact that most cell penetrating peptides (CPPs) have multiple arginine residues [12,13]. Increasing in the number of cationic residues in polymers and dendrimers, or on the surface of nanoparticles usually makes them more efficient in intracellular delivery; however, cytotoxicity usually increases proportionally to the delivery efficiency [[22], [23], [24]]. Indeed, the cationic **R**_**6**_ peptidesome was found to be highly toxic to cells (Fig. 3a).

To alleviate the toxicity, we devised a coassembly strategy in which the cationic **R**_**6**_ is mixed with a zwitterionic RGD-containing building block (**RGD**_**2**_) during the peptidesome fabrication (Fig. S1). Six arginine residues in **R**_**6**_ were replaced by a peptide sequence of equal length (six amino acids), RGDRGD, to promote efficient coassembly. As anticipated, the zwitterionic **RGD**_**2**_ peptidesome was significantly less toxic to cells than the **R**_**6**_ peptidesome (Fig. 3a). Based on the results, we fabricated coassembled peptidesomes with varying proportions of **R**_**6**_ and **RGD**_**2**_, anticipating that the cytotoxicity and intracellular delivery efficiency of the coassembled peptidesome could be controlled by the **R**_**6**_:**RGD**_**2**_ ratios. For preventing larger aggregate caused by **Pa** encapsulation, we determined the proper drug loading range of the peptidesome by analyzing the fluorescence spectra depending on **Pa** loading concentration (Fig. S9). The spectrum of Peptidesome←**Pa** with 25 ​mol% of **Pa** started to blue-shift (674 ​nm → 698 ​nm) and fully shifted in the spectrum of 50 ​mol%, verifying the fact that **Pa** formed J-aggregate at the certain loading concentration. Even though **Pa** loading capacity seemed to be over 200 ​mol% given that peptidesome←**Pa** with 400 ​mol% of **Pa** started to precipitate, we decided to load **Pa** into peptidesome with under 25 ​mol% for further experiment in order to avoid vesicle elongation caused by J-aggregation of **Pa** as shown in **R**_**6**_ peptidesome (Fig. 2a). Drug loading efficacy of the peptidesome←**Pa** was expected to be nearly 100% given that most of **Pa** would locate within the hydrophobic bilayer of peptidesome due to extremely low solubility of **Pa** in water. Among the various ratios tested, a peptidesome with **R**_**6**_:**RGD**_**2**_ ​= ​1:9 was found to be both nearly nontoxic (Fig. 3b). As shown in TEM data, the morphology of the peptidesome maintained the vesicular structure after coassembly (Fig. 3c and d). The range of particle size was 149.6 ​± ​70.6 ​nm and the thickness of bilayer was 10.86 ​± ​2.1 ​nm which well matched with the schematical structure of non-interdigitated bilayer model (∼10.35 ​nm). We further confirmed that the overall vesicular structure of peptidesome was not modified by encapsulation of **Pa** (Fig. 3e). However, the size of peptidesome←**Pa** (**Pa**-loaded peptidesome) somehow decreased to 113 ​± ​68.5 ​nm. Referring the AFM data and fluorescence spectra, we modeled peptidesome←**Pa** (Fig. 3f). The peptidesome was found to be highly efficient in the intracellular delivery of **Pa** (Fig. 3g and Fig. S10). Minimal colocalization with LysoTracker indicates that most of the **R**_**6**_:**RGD**_**2**_ (1:9) peptidesomes enter the cell via the direct penetration mechanism and therefore are not trapped in endosomes. In summary, the coassembly strategy enabled the selection of the **R**_**6**_:**RGD**_**2**_ (1:9) peptidesome, which is nearly nontoxic while simultaneously mediating high-efficiency delivery primarily to the cytosol and even to the nucleus. Moreover, peptidesomes containing the RGD sequence are expected to have tumor-targeting capability via RGD-integrin interactions [25,26] and better *in vivo* performance due to the zwitterionic character of RGD [27].

### 3.4 Prevention of large aggregate formation under *in vivo* conditions

**Fig. 4:**
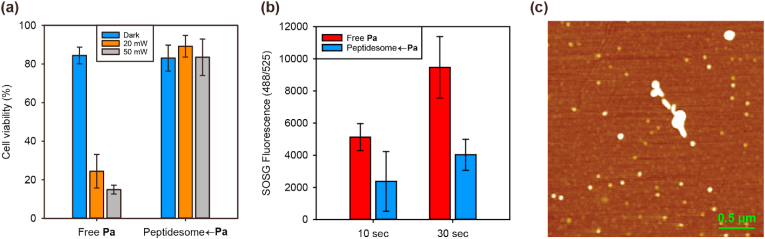
Nonspecific aggregation of peptidesomes under physiological conditions. (a) PDT effect in SCC7 cells. (b) Quantification of singlet oxygen with Singlet Oxygen Sensor Green (SOSG). The samples were dissolved in RPMI 1640 medium containing 10% foetal bovine serum, and IR laser irradiation was performed for the indicated times. (c) AFM image of the peptidesome←**Pa** in RPMI 1640 medium (without the serum). **R**_**6**_:**RGD**_**2**_ ​= ​1:9.

Having controlled the nano- and biostructural properties of the peptidesomes in terms of intracellular delivery efficiency and cytotoxicity, we then evaluated their tumoricidal photodynamic effects in tissue culture. Squamous cell carcinoma 7 (SCC7) cells were treated with free **Pa** or the peptidesome←**Pa** for 24 ​h, followed by infrared (IR) laser irradiation (671 ​nm) at 1.59 ​J/cm^2^. Cytotoxicity as a measure of the photodynamic killing of cancer cells was determined using the MTT assay. The cell viability irradiated with the laser only was about 97.6%, which means that it did not significantly affect cell survival (Fig. S11). As shown in Fig. 4a, there was essentially no PDT effect on cells treated with the peptidesome←**Pa**, in contrast to the strong dose-dependent PDT effect of free **Pa**. To probe the reason behind this negative result, the amount of single oxygen (SO) generated was quantified after the incubation of both groups in cell culture medium (with serum). The amount of SO was far higher for free **Pa** than for peptidesome←**Pa**, which accounts for the results of the PDT assay (Fig. 4b). It has been shown that the aggregation of porphyrin derivatives reduced SO generation and negatively affected PDT efficacy [28]. As a model of physiological conditions, investigation of the nanostructural state in cell culture medium (without serum) showed the coexistence of large aggregates with discrete (not aggregated) peptidesomes (Fig. 4c). The aggregation would have been more severe in cell culture medium containing serum. Taken together, the nonspecific aggregation of the peptidesome←**Pa** is likely responsible for the reduced SO generation and the lack of PDT effect. The morphology of peptidesome before and after NIR irradiation was also monitored by AFM (Fig. S12). Although the peptidesomes were more likely to deform as the size became larger, the overall size distribution and vesicular structure seemed to be maintained to some degree.

DLS investigation reconfirmed the severe aggregation of the peptidesome←**Pa** in solutions containing a physiologically relevant amount of salt and buffer (e.g., PBS), serum-free medium, and 0.9% saline (Table 1). It is expected that the aggregation would become more severe in the presence of serum. Electrostatic screening of the nanoparticle charges by salt ions is likely to decrease the colloidal stability, resulting in the formation of large aggregates (coagulation) [29,30]. In addition, the intrinsic propensity of the peptidesome←**Pa** to form elongated superstructures combined with the increased hydrophobic strength of the peptide building block under high ionic strength conditions can account for eventual formation of large aggregates [31,32].

**Table 1:** Size of the peptidesome←**Pa** in various solution conditions.

Solution condition^a^	Diameter (D_h_)^b^
Distilled water (DW)	104 ​nm
Phosphate-buffered saline (PBS)	*ca*. 400–700 ​nm
Serum-free medium	*ca*. 500–1600 ​nm
5% glucose	117 ​nm
5% glucose +0.9% saline	410 ​nm
5% glucose +20% glycerol	100 ​nm
5% glucose +20% glycerol +0.9% saline	310 ​nm

a Peptidesome←**Pa** [**R**_**6**_:**RGD**_**2**_ (1:9) peptidesome loaded with **Pa** (17 ​mol%)].

b *D*_h_ was measured using DLS.

The formation of large aggregates under physiological conditions prompted us to find the optimal solution condition in which the peptidesome←**Pa** maintains a sufficiently small nanostructural state (≤100 ​nm) and the solution ionic strength is compatible with *in vivo* osmolarity and tonicity. Polyols are neutral in charge and, as cosolvents, are known to make protein conformations more compact, thereby inhibiting protein aggregation [33]. Because SPNs are similar to proteins in their constituents, we hypothesized that polyols might increase the colloidal stability of SPNs and help maintain the *in vivo* osmolarity and tonicity of the peptidesome injection. We first considered glucose as a cosolvent. Glucose is a biocompatible molecule, and isotonic 5% glucose has been widely used as an intravenous infusion. The diameter of the peptidesome←**Pa** increased only slightly to 117 ​nm in 5% glucose compared to the nanoparticle in DW (Table 1). Encouraged by the results, we then used glycerol as a second additive to further reduce the nanoparticle size. Glycerol, among many polyols, is one of the most effective protein stabilizers [33]. The diameter of nanoparticles was further reduced to 100 ​nm when 20% glycerol was used in combination with 5% glucose. Thus, polyols can stabilize SPN structures as they stabilize protein structures, and a solution of 5% glucose and 20% glycerol (G&G solution) was found to be suitable for preventing nanoparticle aggregation in *in vivo* applications. To investigate preventing effect of G&G solution, we measured size of the peptidesome←**Pa** (G&G) in 10% (v/v) fetal bovine serum. The peptidesome←**Pa** (G&G) had a peak around 100 ​nm in size without severe aggregation with serum protein (Fig. S13). The peak at 10 ​nm was that of serum protein added.

### 3.5 Necessary conditions for successful *in vivo* therapy with SPN nanodrugs

**Fig. 5:**
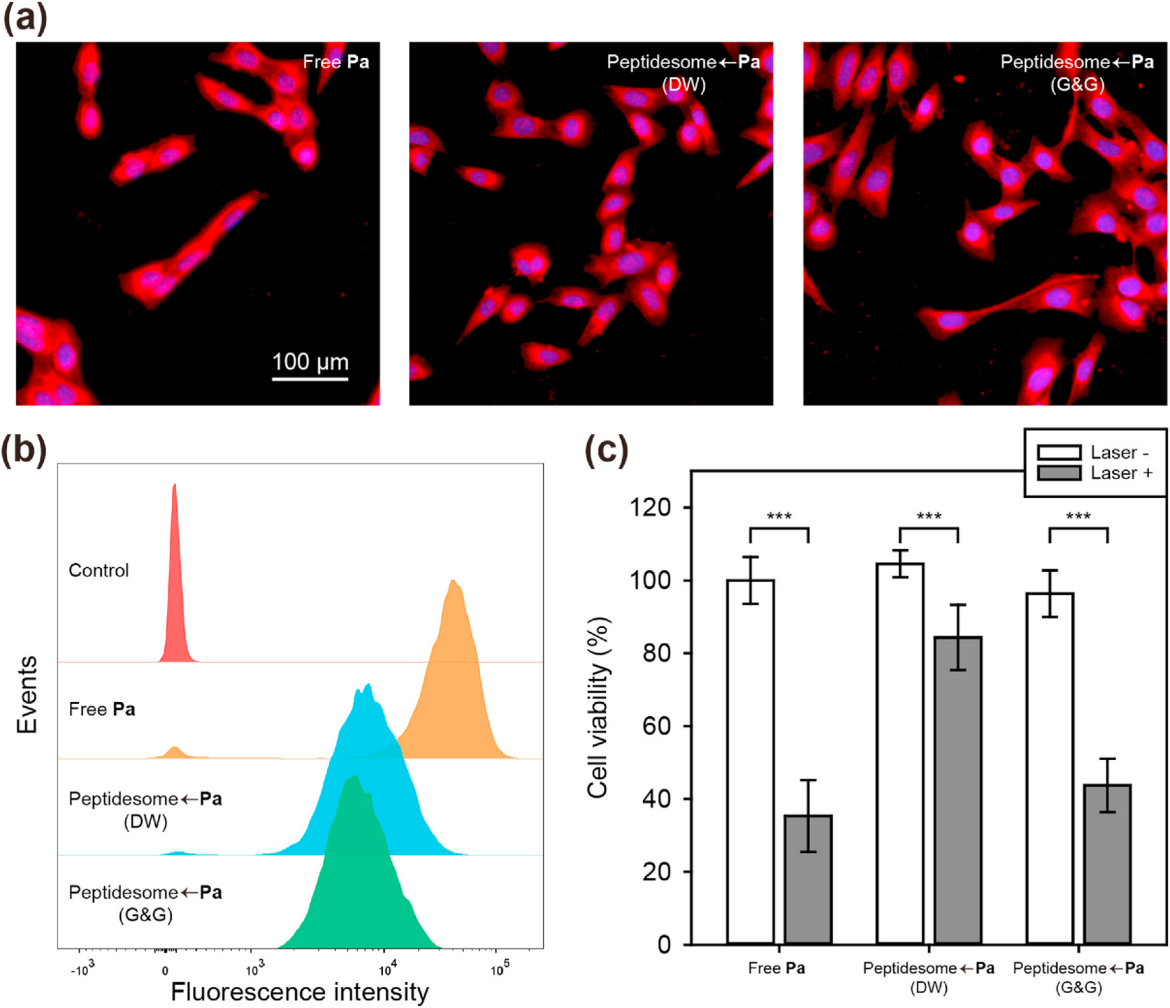
Correlation between the uptake efficiency and PDT effect *in cellulo*. (a) CLSM images of **Pa** (red) in SCC7 cells after 4 ​h of treatment. Blue: nucleus. (b) Flow cytometry analysis of cell uptake. (c) Dark toxicity and PDT against SCC7 cells. Error bar represents mean ​± ​standard deviation (n ​= ​3). Statistics were performed by a two-sample Student's t-test. ∗∗∗*P* ​< ​0.001. (G&G ​= ​5% glucose ​+ ​20% glycerol).

We first validated the effectiveness of the G&G solution in tissue culture. Regarding the cell internalization efficiency, free **Pa** was 5-fold more efficient than peptidesome←**Pa** (Fig. 5a and b). The lipophilicity of free **Pa** should account for its high uptake efficiency. Considering the results described above, significant aggregation is expected to occur when peptidesome←**Pa** (DW) is added to cell culture medium for cell treatment, whereas aggregation would be minimal for peptidesome←**Pa** (G&G). Because the peptidesome←**Pa**s in DW or in the G&G solution showed similar levels of uptake efficiency, the aggregation status may not be a significantly important factor for cell internalization in tissue culture. In contrast, the PDT effect was influenced by the aggregation status (Fig. 5c). The peptidesome←**Pa** (G&G) was approximately 2-fold better than the peptidesome←**Pa** (DW) in terms of PDT effect, indicating that even if cell internalization efficiency is similar, aggregation status does influence the final PDT effect. Furthermore, we investigated PDT-induced ROS generation under *in vitro* and *in vivo* using 2′,7′-dichlorofluorescin diacetate (DCFDA), ROS detection agent. DCFDA fluorescence of peptidesome←**Pa** (G&G)-treated SCC7 cells significantly increased under laser irradiation *in vitro* and *in vivo* compared to control group, suggesting that ROS was well generated by photodynamic effect of peptidesome←**Pa** (G&G) (Fig. S14).

**Fig. 6:**
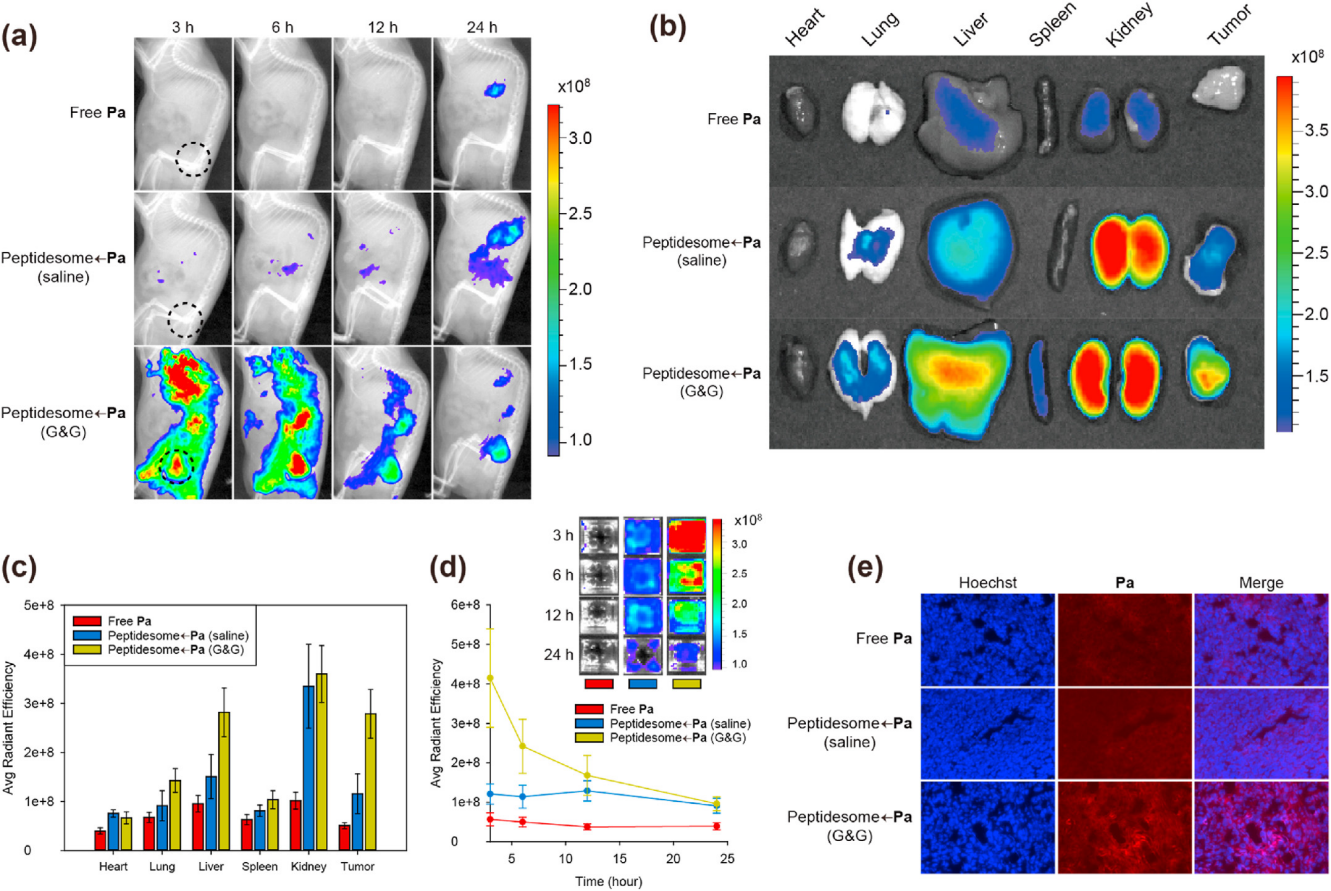
*In vivo* biodistribution of peptidesome←**Pa** in tumor-bearing mice. (a) Time-dependent whole body fluorescence images after intravenous injection of free **Pa** or the peptidesome←**Pa** (2 ​mg/kg of **Pa**). Black dotted circles indicate tumor regions. (b) *Ex vivo* fluorescence image of each organ. (c) Quantification of fluorescence intensity from the data in (b). Error bar represents mean ​± ​standard deviation (n ​= ​3). (d) Blood fluorescence images and quantification data. (n ​= ​3). Blood was obtained from the tail of a mouse at the indicated time points. (e) Fluorescence images of cryosections of tumor tissues excised 24 ​h after the administration of the drugs.

Next, we investigated the effect of nanoparticle aggregation on blood circulation and tumor tissue accumulation in a xenograft mouse model bearing SCC7 cell-derived cancer. After intravenous injection of the drugs, whole-body NIR fluorescence imaging was performed by an IVIS Lumina XRMS system at different time points. Whole-body fluorescence images showed significantly more intense **Pa** fluorescence in the tumor region in the mice treated with the peptidesome←**Pa** (G&G) than in the mice treated with the peptidesome←**Pa** (saline) or free **Pa** (Fig. 6a). Nanoparticles were severely aggregated in the peptidesome←**Pa** (saline) because the peptidesome←**Pa** fabricated in DW was diluted with 0.9% saline to adjust the osmolarity before intravenous injection. *Ex vivo* fluorescence imaging of major organs and tumors obtained 24 ​h postinjection further supports the higher tumor accumulation efficiency of the peptidesome←**Pa** (G&G) than the others (Fig. 6b and c). The fluorescence of **Pa** in blood was also much higher for the peptidesome←**Pa** (G&G) (Fig. 6d). Consistent with the above results, the peptidesome←**Pa** (G&G) showed higher accumulation in tumor tissues than the other treatments (Fig. 6e). The enhanced permeability and retention (EPR) effect [34] and RGD-integrin interactions [35] are likely to have played important roles in the tumor targeting of peptidesome←**Pa**. Taken together, the prevention of SPN aggregation is crucial for high efficiency tumor targeting *in vivo*.

**Fig. 7:**
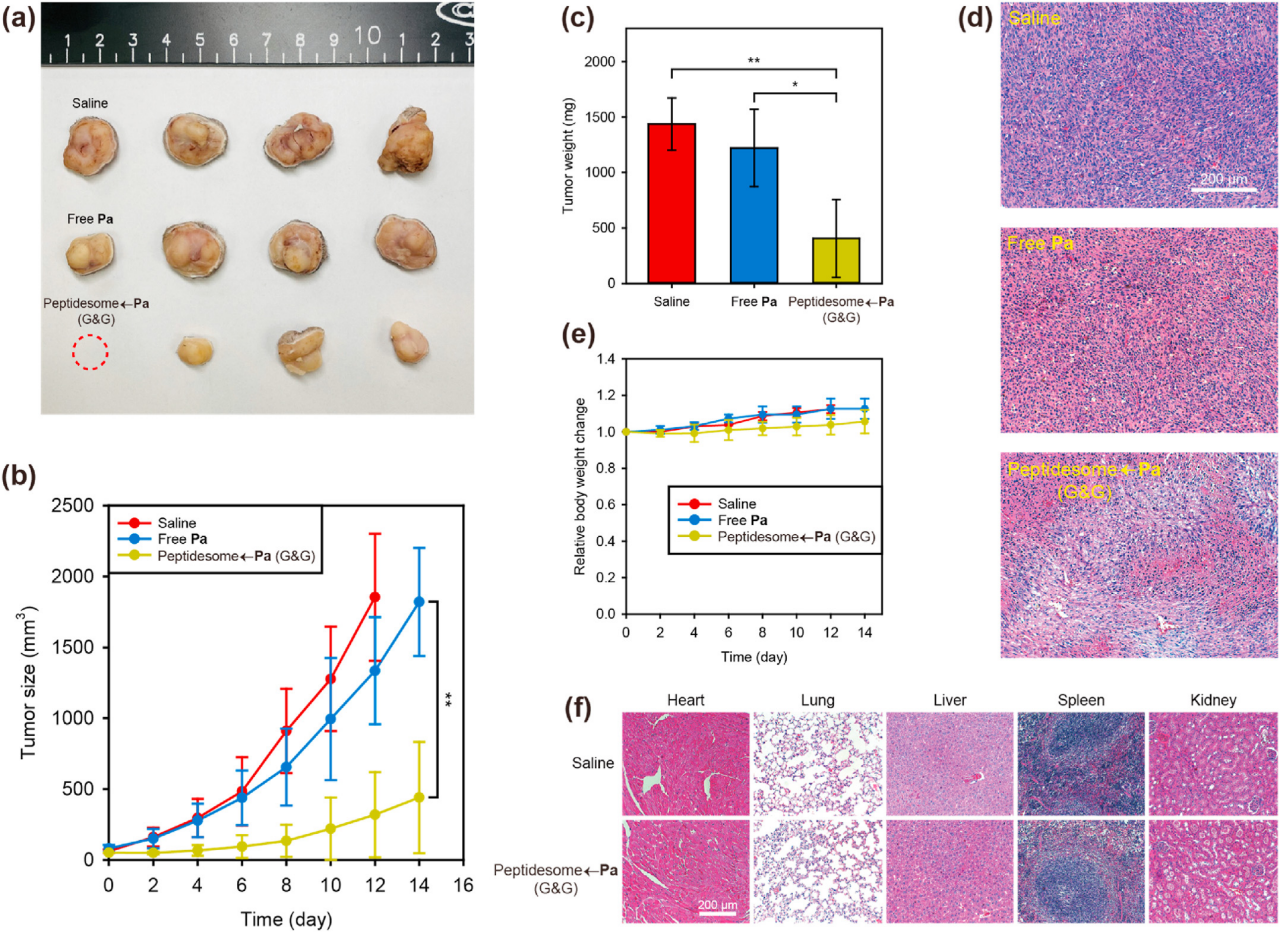
*In vivo* anticancer efficacy against SCC7 tumor-bearing mice. (a) Photographic image of SCC7 tumors excised 14 days after NIR irradiation. A dotted circle (red) indicates the complete regression of the tumor. (b) Quantification of the tumor growth. (c) Excised tumor weight after 14 days. (d) Histological H&E images of tumor tissue. (e) Body weight changes for 14 days. (f) Histological analysis of major organs by H&E staining. Error bar represents mean ​± ​standard deviation (n ​= ​4). Statistics were performed by one-way ANOVA and post hoc Tukey tests in (b). Statistics were performed by a two-sample Student's t-test in (c). ∗*P* ​< ​0.05 and ∗∗*P* ​< ​0.01.

To investigate the photodynamic anticancer efficacy, we intravenously injected the drugs, and 3 ​h later, NIR laser (671 ​nm) irradiation was applied to the tumor region at 0.53 ​W/cm^2^ for 15 ​min under anesthesia. The laser intensity used *in vivo* was set at a level that did not cause damage to the tumor, referring to the previous paper of our group [36]. Peptidesome←**Pa** (G&G) strongly inhibited tumor growth compared to the other groups (Fig. 7a). Because the tumors grew too vigorously, the saline group was euthanized on day 12 due to concerns about animal ethics. At day 14, the tumor size of mice treated with peptidesome←**Pa** (G&G) was 4.2 times smaller than that in mice treated with free **Pa** (Fig. 7b). At the end of the therapy, the weight of the excised tumor for the free **Pa**-treated group (1222 ​mg) was approximately 3 times larger than that of the peptidesome←**Pa** (G&G)-treated group (406 ​mg) (Fig. 7c). During therapy, no significant change was observed in the body weight of all groups, which indicated that the injected drugs had no serious systemic toxicity (Fig. 7d). In H&E images, tumor tissue from the peptidesome←**Pa** (G&G)-treated group showed a severely destroyed structure, which was markedly different from the groups treated with saline or free **Pa** (Fig. 7e). For comparison, similar histological images of major organs (heart, lung, liver, spleen, and kidney) were obtained for all three drug-treated groups, indicating that no significant damage to organs other than the tumor tissue occurred (Fig. 7f).

**Fig. 8:**
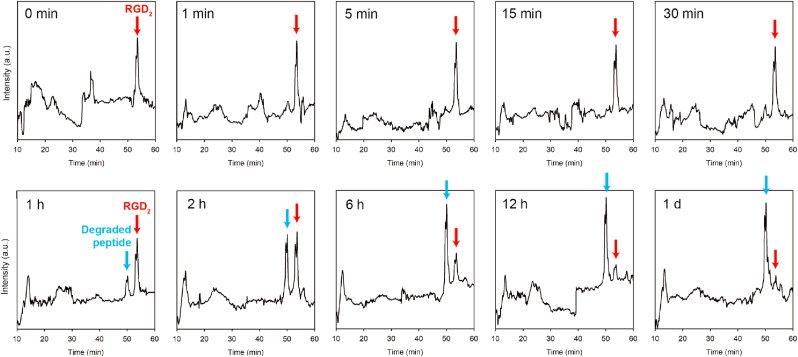
Protease resistance of the peptidesomes. **RGD**_**2**_ peptidesome (50 ​μm, 300 ​μL) was treated with trypsin from bovine pancreas (0.39 ​μg) in PBS and the mixture was incubated at 37 ​°C. At the appropriate time points, aliquots were taken, and the reaction was quenched by the addition of 0.2% TFA (v/v). Before HPLC analysis, acetonitrile was added to the final concentration of 50% (v/v) to disrupt the molecular assembly. The reaction mixture was then analyzed with reverse-phase HPLC using a C4 column.

In general, one of the most fatal weaknesses of peptides as drugs is rapid proteolytic degradation *in vivo* [37]. The peptidesomes developed in this study maintained a high level of resistance to proteolytic degradation, as demonstrated by the *in vivo* performance and the protease-mediated *in vitro* degradation experiments (Fig. 8). The formation of tight molecular assemblies is likely responsible for the high proteolytic stability of the peptidesomes. Taking all the results together, the morphology and size control of nanoparticles, the control of cytotoxicity, the improvement in cell uptake efficiency, the installation of endosome escape function, the protection from proteolytic degradation through self-assembly, and the prevention of nonspecific aggregation under physiological conditions are all the *necessary conditions*, although they may not be sufficient, for high-efficiency tumor targeting and anticancer therapy of SPNs *in vivo*.

## 4 Discussion

**Fig. 9:**
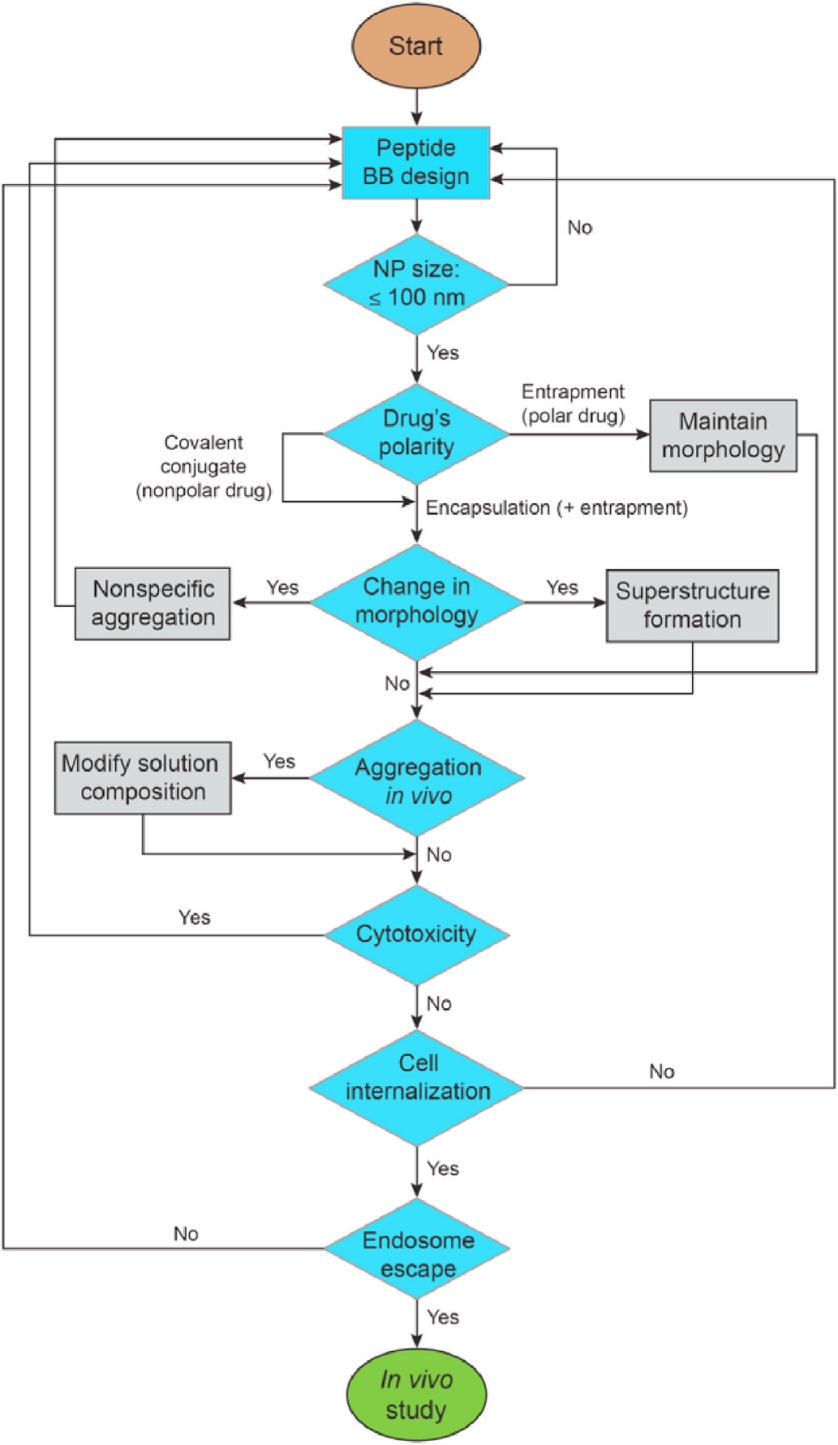
Critical points and necessary conditions for the successful correlation of *in vitro* and *in vivo* studies during SPN nanodrug development. BB: building block. NP: nanoparticle.

Peptides as building blocks have a number of unique characteristics that distinguish them from lipids and synthetic polymers. A high propensity to form hydrogen bonds imposes directionality during the self-assembly process, chiral amino acids impart handedness, and polypeptide chain flexibility is restricted by the planarity of the peptide bond. Thus, many unprecedented challenges can be encountered during the translation of *in vitro* results obtained from SPN nanodrugs to *in cellulo* and *in vivo* studies and even to clinical applications. Indeed, we encountered many unexpected problems over the course of translating the nanobiostructural properties of SPN nanodrugs obtained *in vitro* to successful *in cellulo* and *in vivo* therapy results. Based on our problem-solving strategies, we summarize the most critical points in SPN nanodrug development as a flowchart (Fig. 9).

The initial step is the design of peptide building blocks. Some brief guidelines are to make them amphiphilic, to consider the potential of hydrogen bond formation, to be aware of the propensity of amino acids to form certain secondary structures, and to choose certain molecular topologies appropriate for the desired nanostructural properties. If the desired morphology is the vesicle, the use of cyclic peptides can be one of the primary options. The second step is the controlled formation of SPNs in appropriate aqueous solution conditions. Solution conditions such as ionic strength, pH, and the presence of cosolvents and the method of sample preparation to control thermodynamic or kinetic pathways can affect nanostructural properties such as morphology and size. Because nanodrugs are usually the most effective at sizes smaller than 100 ​nm, redesign of the building blocks might be required if this condition is not met. Third, we should be aware that the shape and stability of SPNs can be altered after the drug loading process. Fourth, the probability of nanoparticle aggregation increases as the solution conditions become more similar to *in vivo* conditions. Increases in ionic strength and the presence of serum proteins usually decrease the colloidal stability of the nanoparticles. Many *in vitro* studies on self-assembly reported in the literature have been performed in DW or in solutions containing organic solvents. It should be noted that the nanostructural properties obtained from such solution conditions may not be reproduced *in cellulo* and *in vivo*. If we fail to obtain the desired nanostructural properties after all attempts, we probably need to begin again with the design of peptide building blocks. Fifth, the cytotoxicity of SPNs themselves needs to be minimal because nanoparticles are only carriers to transport bioactive drugs to intracellular compartments. Sixth, cell internalization efficiency either by endocytosis or by a direct penetration mechanism should be high enough for the drugs to exert their bioactivities. Seventh, nanoparticles should be able to cross multiple biological barriers, including one of the most important barriers, endosomes. These critical points can be considered minimum requirements. The sequence in the flowchart might be reordered depending on the circumstances.

Some of the important conclusions drawn from this study include the following: (1) the morphology and size of self-assembled peptide nanodrugs can be significantly different *in vitro* and *in vivo*; (2) in tissue culture experiments, cell uptake efficiency does not significantly depend on the size of nanoparticles; (3) even if the overall cell uptake efficiency in tissue culture might be similar, the eventual bioactivity is dependent on the nanoparticle size and the aggregation status; and (4) *in vivo* (in animal) tumor targeting and therapeutic effects are critically dependent on the size of nanoparticles.

In addition to being biocompatible, an additional advantage of peptide building blocks includes the possibility of incorporating cell targeting and penetration functions as a part of the building block without the need to chemically conjugate additional ligand moieties. Our study demonstrates that highly efficient and proteolytically stable SPN nanodrugs for the PDT of solid tumors can be developed through the multivariate control of peptidesomes. Therefore, this study has laid the foundation for the further clinical translation of self-assembled peptide nanodrugs based on peptidesomes.

## Data availability

All experimental data within the article are available from the corresponding author upon reasonable request.

## Declaration of competing interest

The authors declare the following financial interests/personal relationships which may be considered as potential competing interests: Yong-beom Lim has patent pending to 10.13039/501100002573Yonsei University, Office of research affairs/University industry foundation.

## Appendix A Supplementary data

The Supplementary Material is available free of charge at https:// Characterization of peptide synthesized. Additional AFM, CD, and UV-Vis data (PDF).
