# Supplementary material for: Structural control of self-assembled peptide nanostructures to develop peptide vesicles for photodynamic therapy of cancer
Source: Mater Today Bio. 2022 Jun 22;16:100337. doi: 10.1016/j.mtbio.2022.100337 (PMC9254122; doi:10.1016/j.mtbio.2022.100337)
Supplement: Multimedia component 1 [file mmc1.docx]

**Supplementary Material**

**Structural control of self-assembled peptide nanostructures to develop peptide vesicles for photodynamic therapy of cancer**

Soo hyun Kwon^1,†^, Donghyun Lee^2,†^, Hyoseok Kim^1,†^ , You-jin Jung^1^, Heebeom Koo^2^*, and Yong-beom Lim^1^*

^1^Department of Materials Science and Engineering, Yonsei University, Seoul 03722, Republic of Korea

^2^Department of Medical Life Sciences and Department of Biomedicine & Health Sciences, College of Medicine, The Catholic University of Korea, Seoul 06591, Republic of Korea

^†^These authors contributed equally

*E-mail: hbkoo@catholic.ac.kr, yblim@yonsei.ac.kr


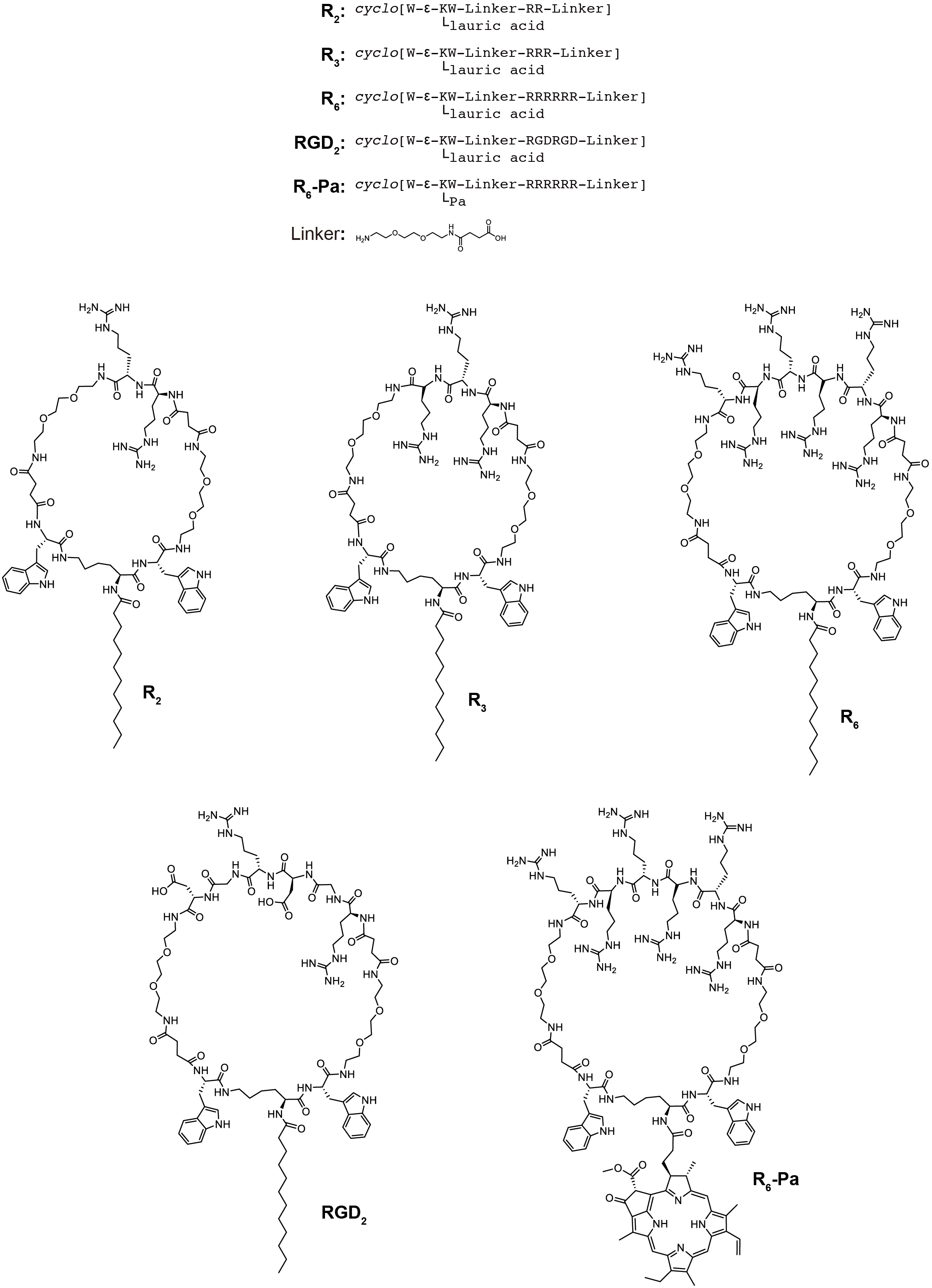


**Fig. S1.** Sequences and chemical structures of cyclic peptide building blocks.


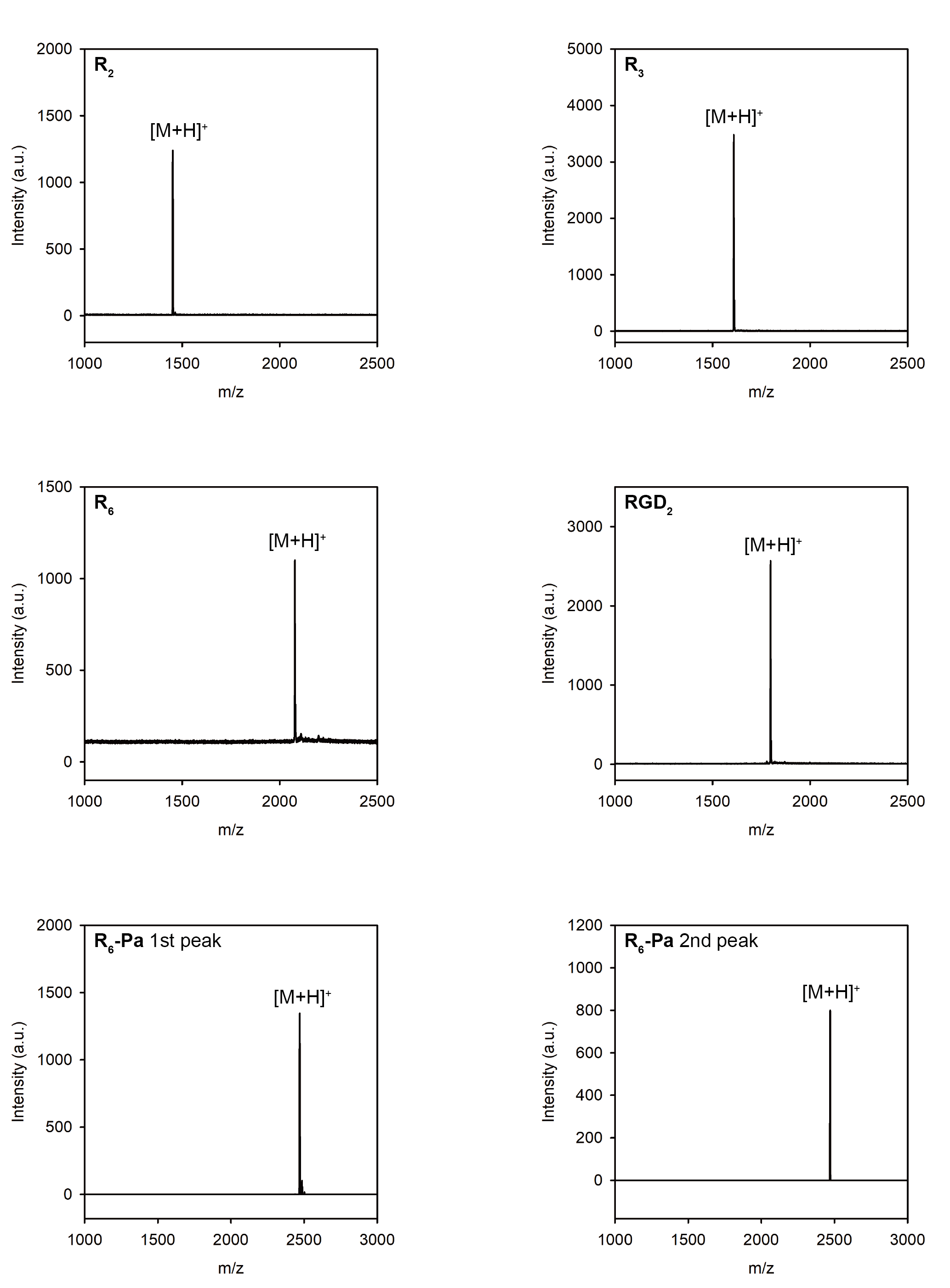


**Fig. S2.** MALDI-TOF MS spectra for the purified the cyclic peptide building blocks.


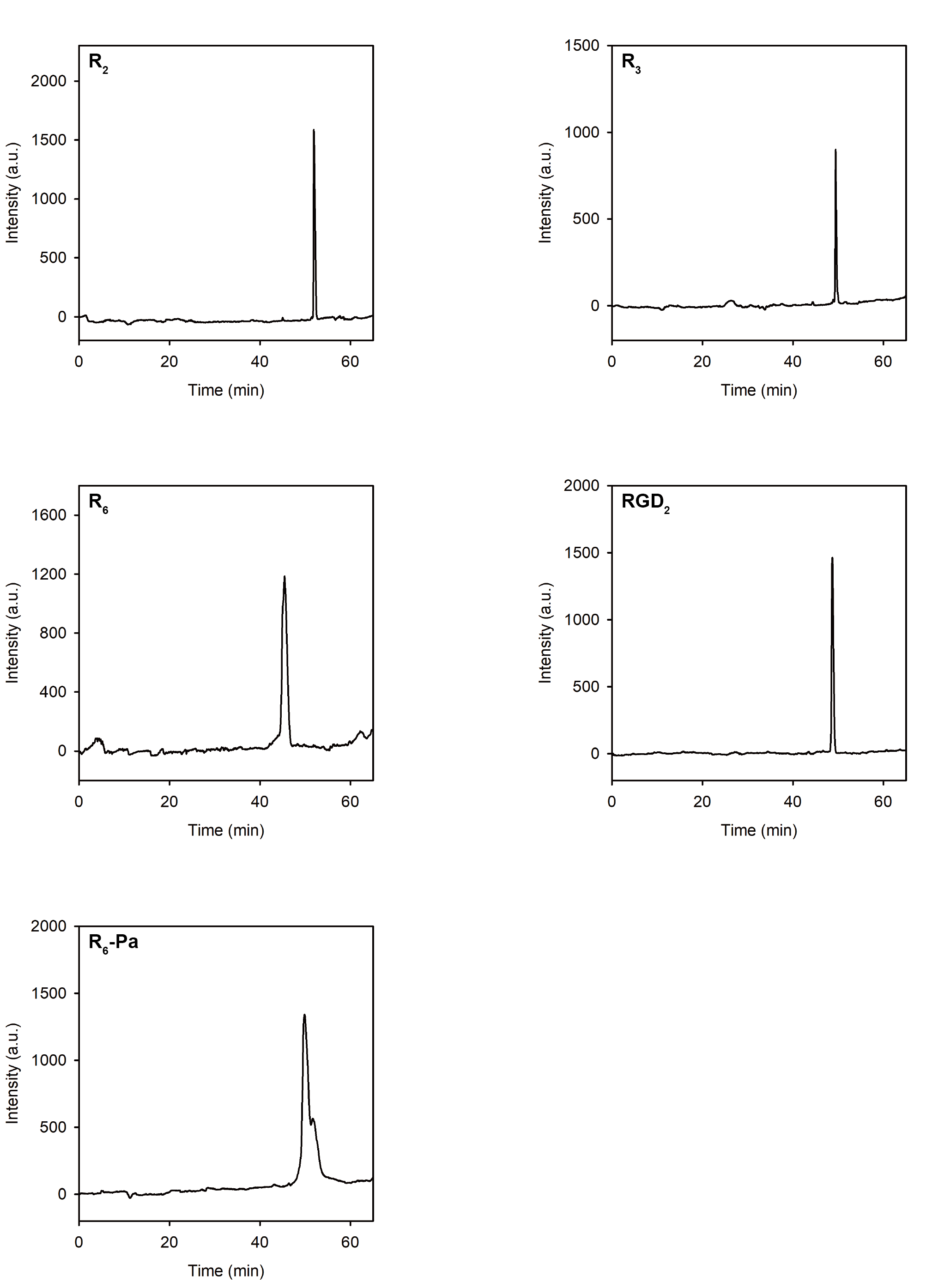


**Fig. S3.** HPLC chromatograms for the purified the cyclic peptide building blocks. The first and second peaks in **R_6_-Pa** are from the same compounds possibly in different conformations (see Fig. S2).


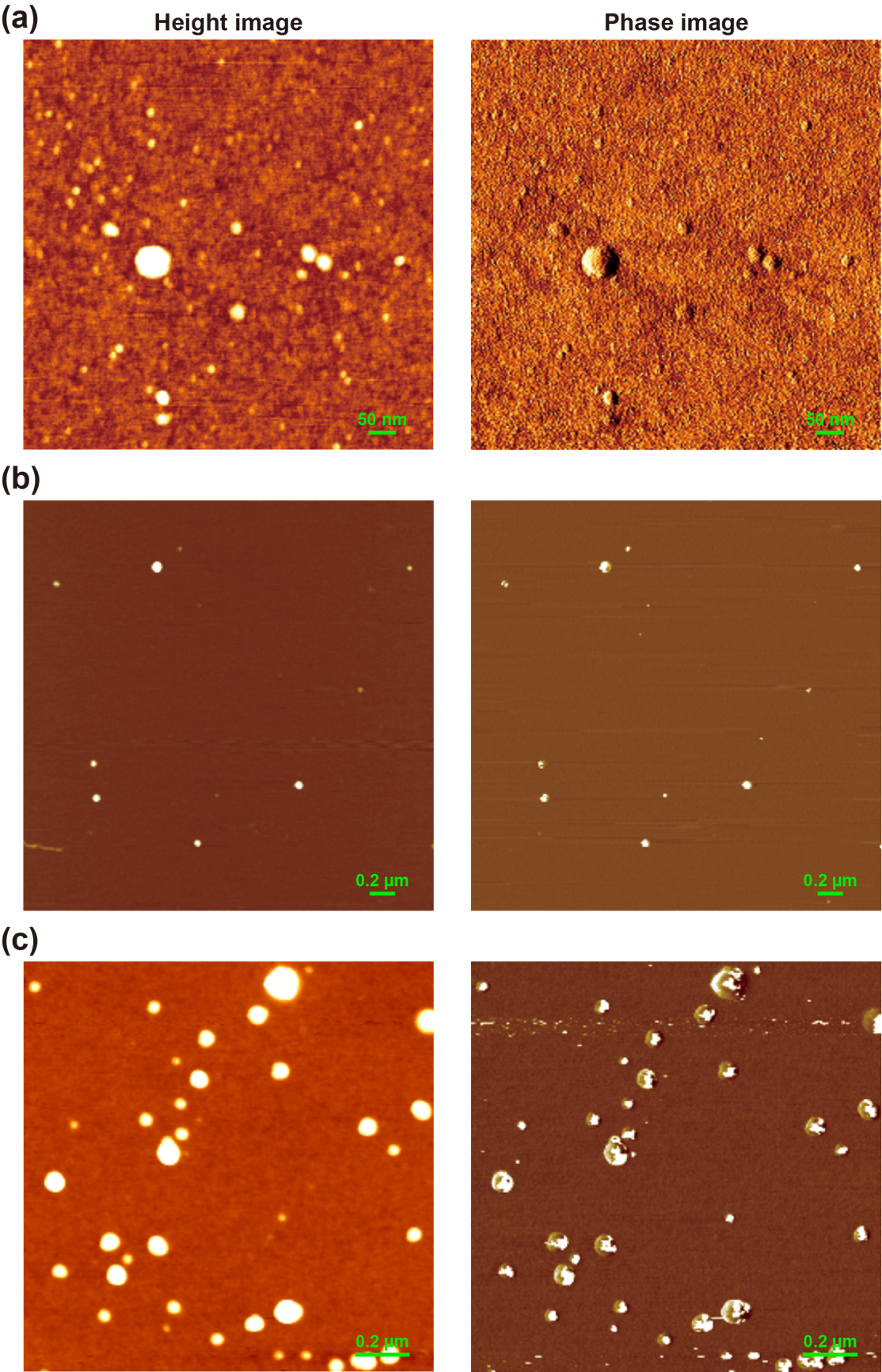


**Fig. S4.** AFM investigation of (a) **R_2_**, (b) **R_3_** and (c) **R_6_** peptidesomes on mica surface.

**
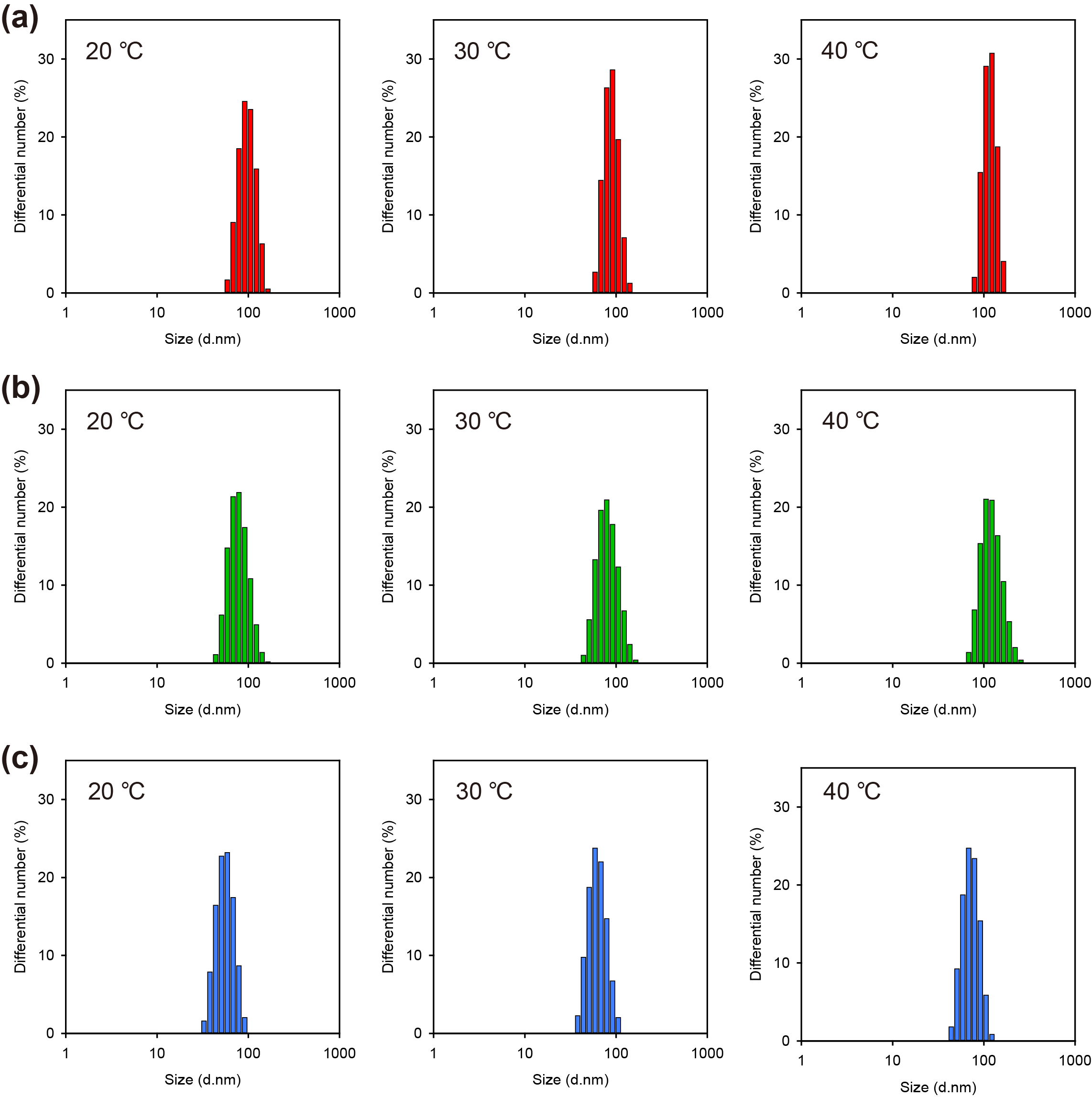
**

**Fig. S5.** Size distribution of (a) **R_2_**, (b) **R_3_** and (c) **R_6_** peptidesome depending on temperature using DLS.

**
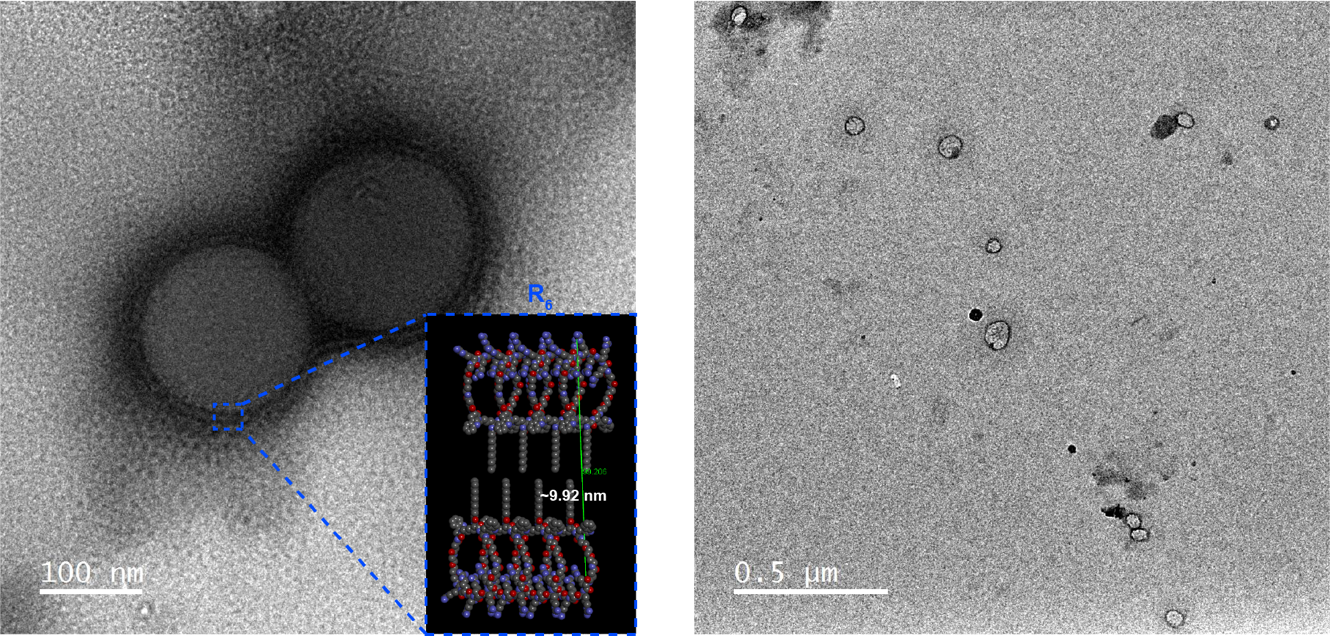
**

**Fig. S6.** TEM investigation of **R_6_** peptidesomes on carbon grid with schematical structure of **R_6_**.


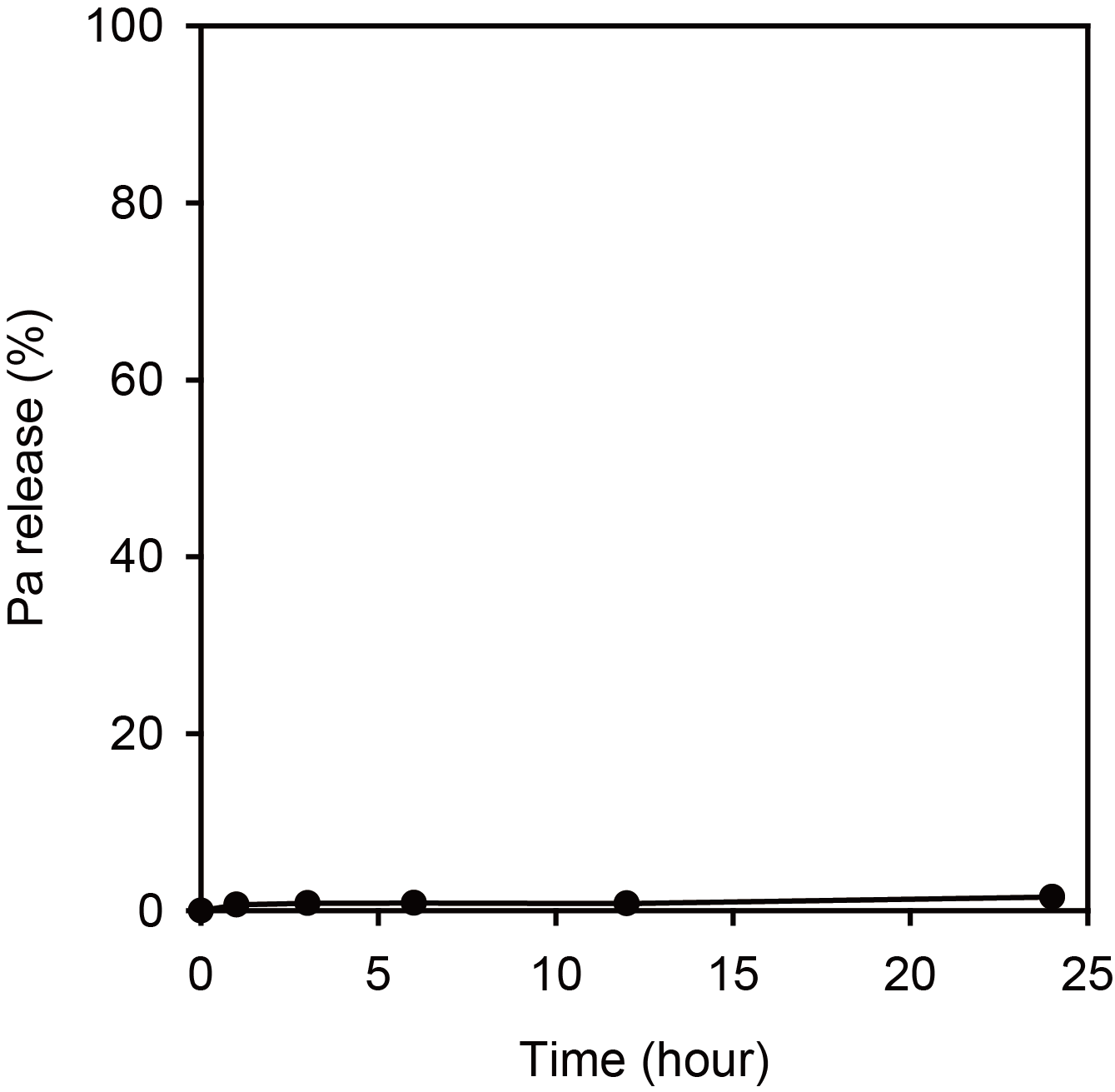


**Fig. S7.** In vitro release of **Pa** from peptidosome.


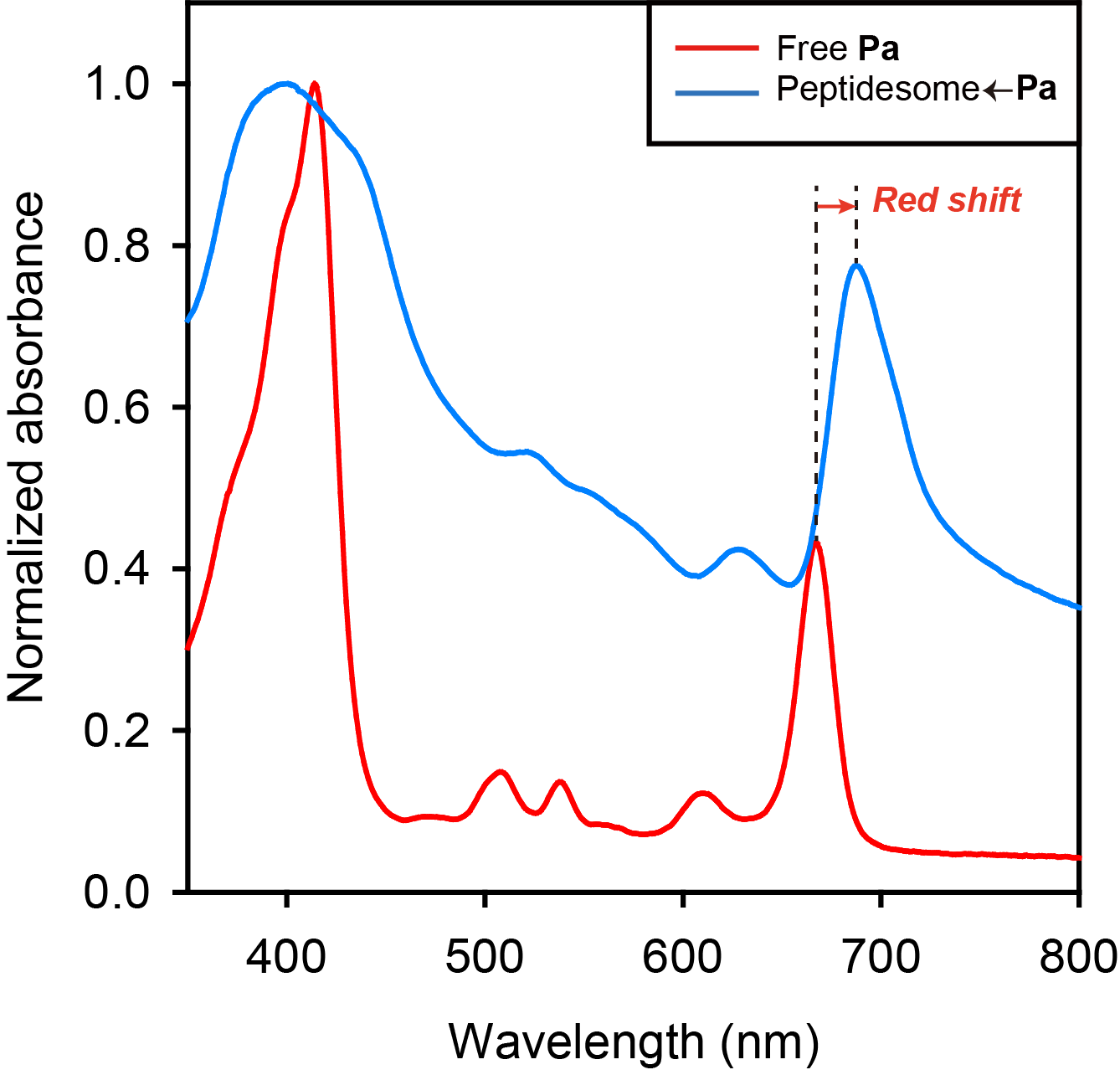


**Fig. S8.** UV absorption spectra. Free **Pa** was dissolved in DMSO. Peptidesome←**Pa** was fabricated in pure water. Peptidesome←**Pa** = **R_6_**:**RGD_2_** (1:9).

**
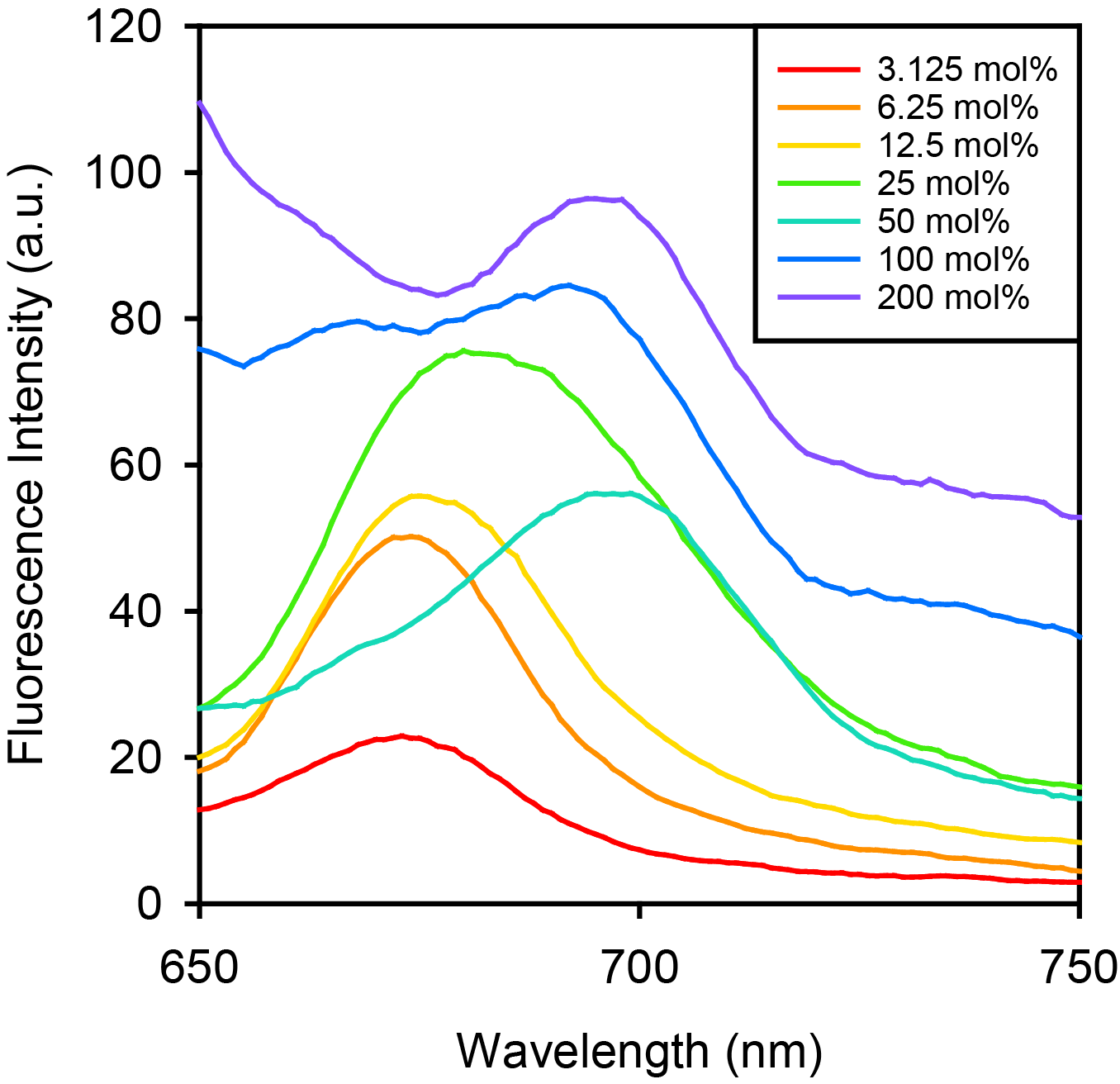
**

**Fig. S9.** Fluorescence spectra of peptidesome←**Pa** depending on **Pa** mol%. Excitation wavelength was 507 nm. Peptidesome←**Pa** = **R_6_**:**RGD_2_** (1:9).

**
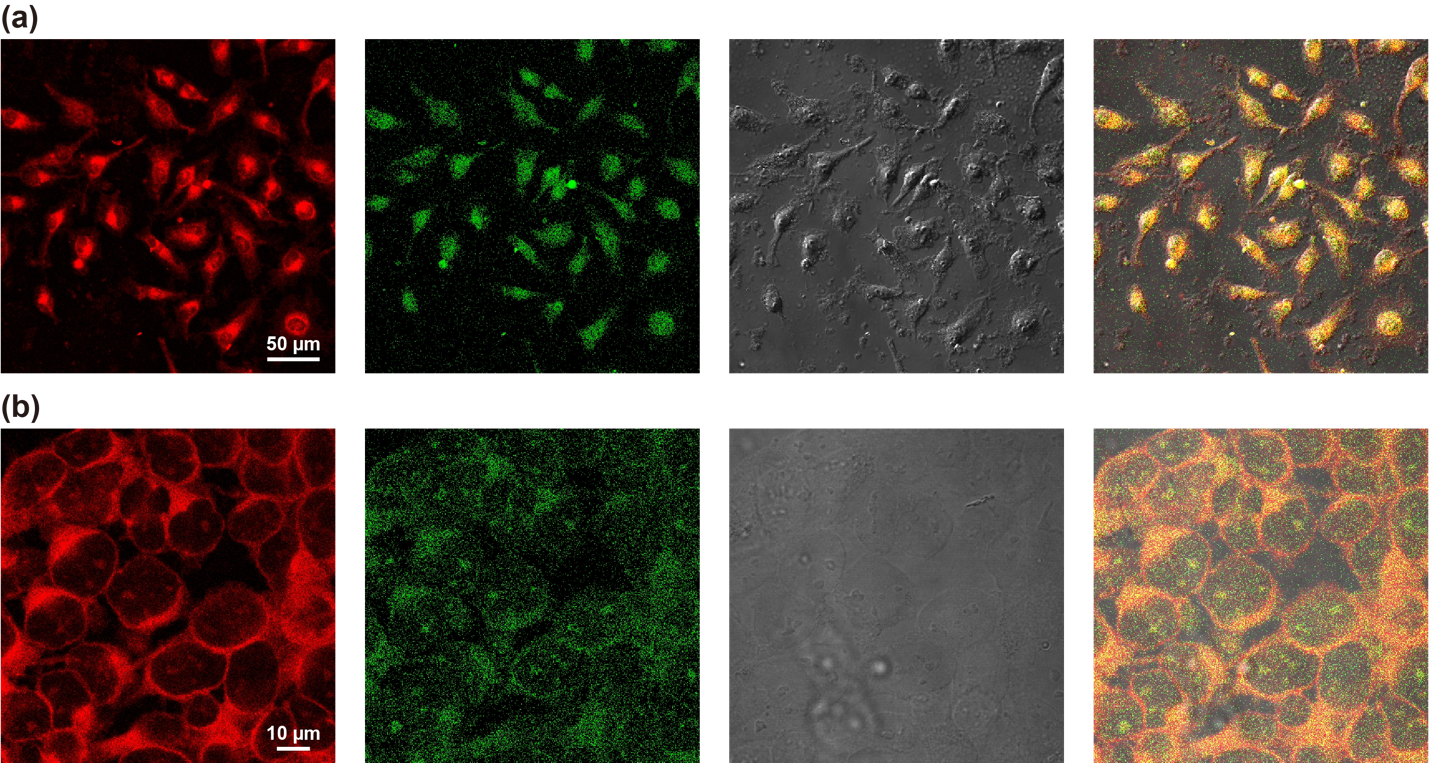
**

**Fig. S10.** Confocal laser scanning microscopy (CLSM) images after the treatment of HeLa cells with the peptidesome←**Pa**. Right: **Pa** fluorescence (red). Middle right: LysoTracker (green). Middle left: Bright field. Left: Overlay of fluorescence from **Pa** (red) and LysoTracker (green) merged with bright field image.


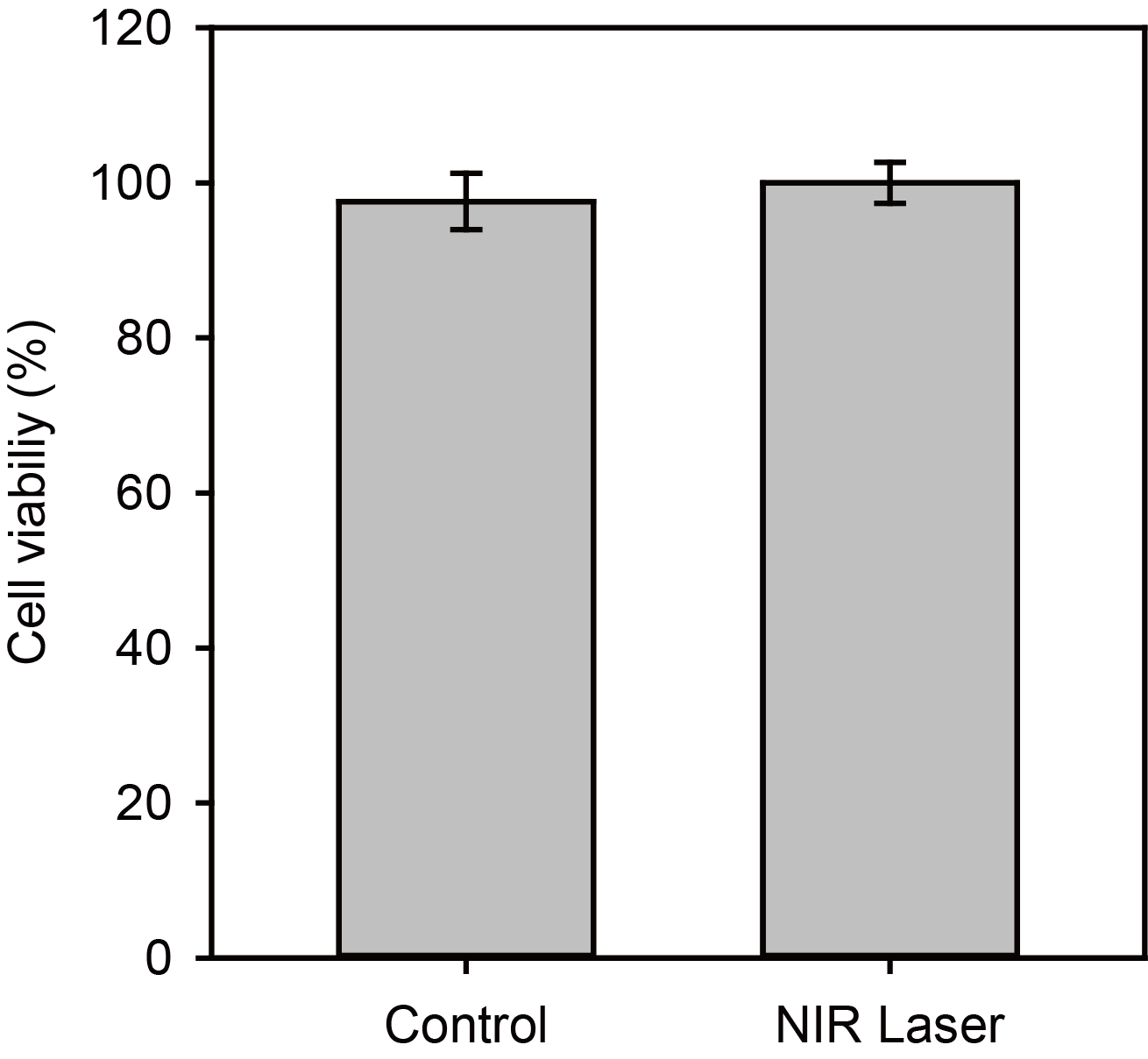


**Fig. S11.** SCC7 cell viability data after laser irradiation.


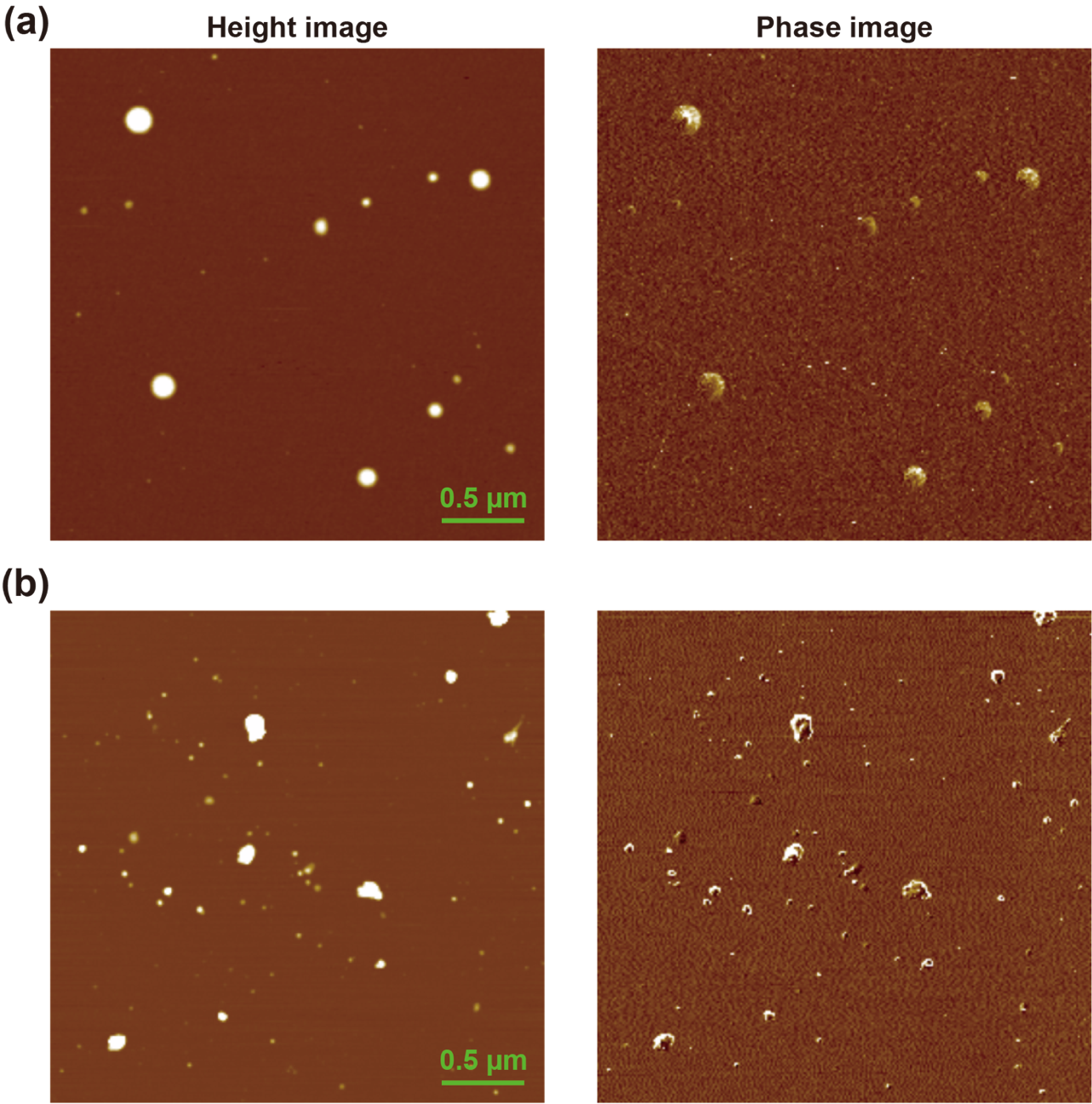


**Fig. S12.** AFM investigation of peptidesome←**Pa** (a) before and (b) after NIR irradiation**.** Peptidesome←**Pa** = **R_6_**:**RGD_2_** (1:9).


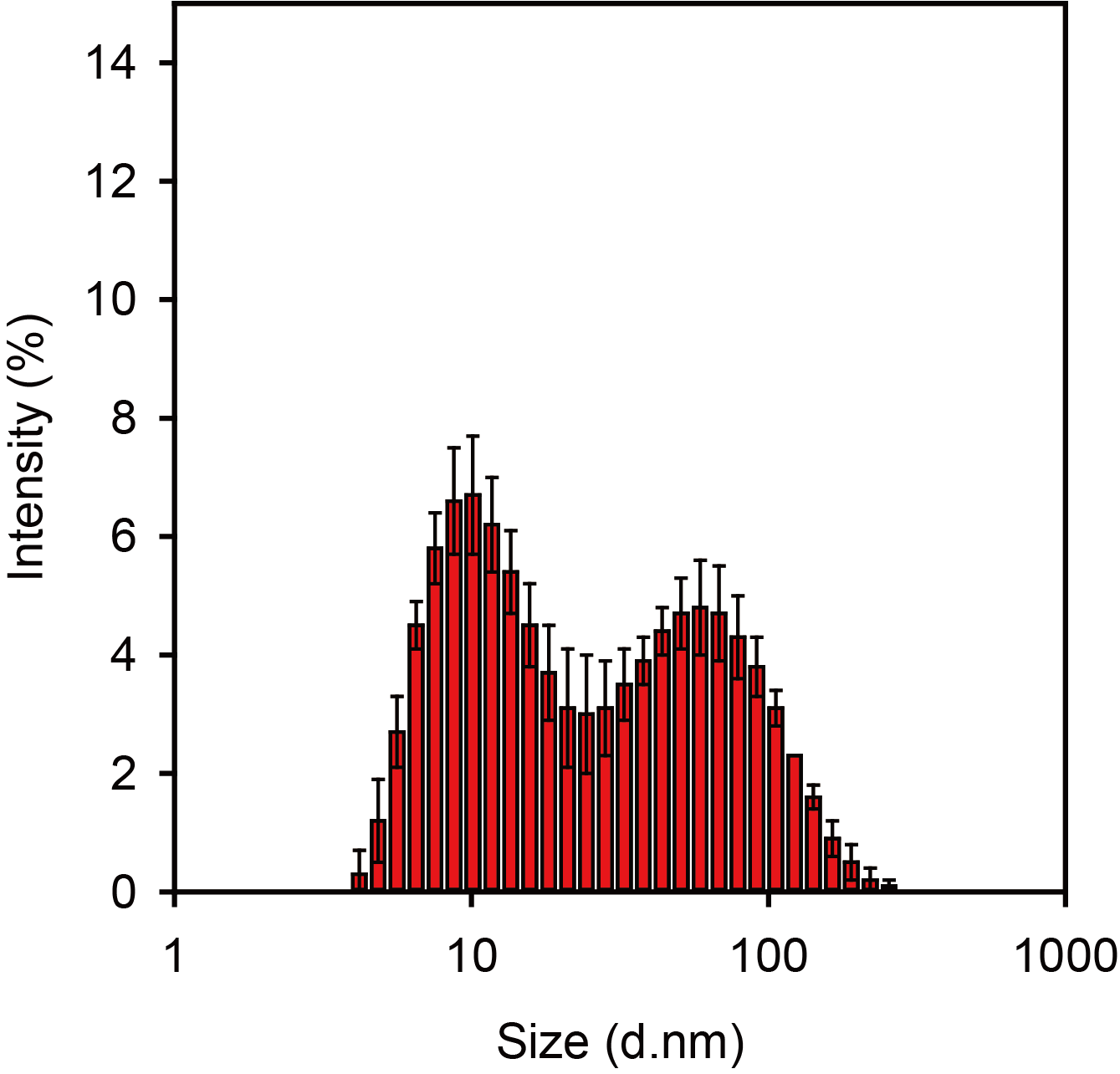


**Fig. S13.** Size distribution of peptidesome←**Pa** (G&G) after mixing fetal bovine serum.


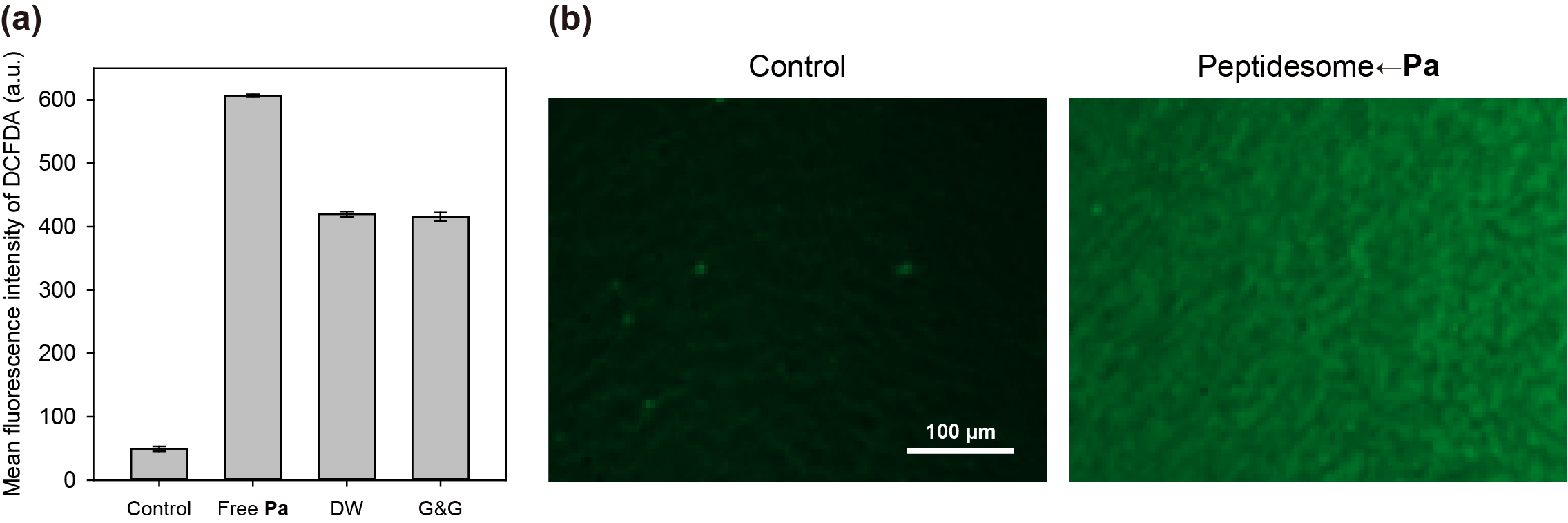


**Fig. S14.** Investigation of ROS generation by PDT using 2’,7’-dichlorofluorescin diacetate (DCFDA). (a) In vitro detection of PDT-induced ROS generation in flow cytometry after treatment of peptidesome in SCC7 cells. Error bar represents mean ± standard deviation (n = 3). (b) In vivo analysis of ROS generation (Green) by PDT in cryosectioned tumor tissue. DW: peptidesome←**Pa** (DW). G&G: peptidesome←**Pa** (G&G).
